# BatGPT-Chem: A Foundation Large Model for Chemical Engineering

**DOI:** 10.34133/research.0827

**Published:** 2025-09-10

**Authors:** Yifei Yang, Runhan Shi, Zuchao Li, Shu Jiang, Bao-Liang Lu, Qibin Zhao, Yang Yang, Hai Zhao

**Affiliations:** ^1^ School of Computer Science, Shanghai Jiao Tong University, Shanghai 200240, China.; ^2^ Key Laboratory of Shanghai Education Commission for Intelligent Interaction and Cognitive Engineering, Shanghai Jiao Tong University, Shanghai 200240, China.; ^3^ Shanghai Key Laboratory of Trusted Data Circulation and Governance in Web3, Shanghai, China.; ^4^ School of Artificial Intelligence, Wuhan University, Wuhan 430070, China.; ^5^ School of Artificial Intelligence and Computer Science, Nantong University, Nantong 226019, China.; ^6^ RIKEN Center for Advanced Intelligence Project, Tokyo 103-0027, Japan.

## Abstract

Large language models (LLMs) have showcased remarkable capabilities in the realm of AI for Science, and chemistry has greatly benefited from the advancement of AI tools. With a strong capacity for learning sequential data like natural language, LLMs offer immense potential. Despite this promise, the application of LLMs in chemistry remains limited, with few models specifically designed for chemical data and tasks. Hence, we propose leveraging LLMs to comprehensively model both chemical sequences and natural language sequences, aiming to tackle diverse chemical tasks. We introduce BatGPT-Chem, a general foundation large-scale model with 15 billion parameters tailored for chemical engineering. Built on a corpus of over 100 million chemical instances, BatGPT-Chem specializes in 5 core tasks: retrosynthesis prediction, molecule design, molecule description, product inference, and yield prediction. BatGPT-Chem comprehensively models the information flow between chemical language and natural language, enabling full-spectrum prediction across chemical tasks. It is one of the largest bilingual chemistry-specific LLMs, supporting both English and Chinese for input and output. BatGPT-Chem is also the first automated retrosynthesis tool capable of explicitly predicting reaction conditions, a critical but often overlooked aspect in previous models. Through rigorous zero-shot evaluations, BatGPT-Chem demonstrates state-of-the-art performance, surpassing both existing chemical LLMs and general-purpose models in accuracy and validity across a diverse range of tasks. Notably, it demonstrates superior ability in predicting both reactants and reaction conditions, as well as strong generalization in low-data settings. These results suggest that BatGPT-Chem is among the most advanced and practical chemical LLMs, with strong potential to support real-world applications in synthesis planning, drug discovery, and materials design.

## Introduction

The continuous advancement of artificial intelligence (AI), particularly large language models (LLMs), has empowered machines to excel in various domains [1,2]. Natural language serves as the most intuitive means of communication and expression, rendering the path for AI applications remarkably smooth as long as effective natural language understanding is achieved. The high intelligence of LLMs is regarded as a key element in overcoming the “AI4Science” challenge in recent years.

The rapid advancement of the field of chemistry is inseparable from the progress in computer-assisted synthesis technology and automated management of chemical knowledge. In the past few decades, various computer-assisted synthesis algorithms or software based on reaction templates have been proposed, such as LHASA [3], SECS [4], and SYNLMA [5]. However, these reaction templates are expert-developed manual rules, which not only incur important manpower and time costs but also fail to cover all complex organic chemistry prediction problems. Databases such as Reaxys (https://www.reaxys.com/), SciFinder (https://scifinder-n.cas.org/), ChemSpider (http://www.chemspider.com/), and ChASe (https://www.psds.ac.uk/chase) have assisted chemists in searching for literature sources or similar reaction instances. However, the full potential of modern computers has not yet been fully realized, as reaction space searches still require manual intervention by chemists. Subsequently, a multitude of neural network-driven chemical prediction algorithms such as HAQC [6], the reaction fingerprinting-based neural network system [7], Chemoton [8], Text2Mol [9], Egret [10], a merged hypergraph neural network-based method [11], a general multiplicative Zagreb indices-based algorithm [12], and the symmetric division Szeged index [13] have emerged, contributing to the advancement of AI in the field of chemistry. However, these methods often specialize in completing specific chemical tasks, such as singular product prediction or molecular description. Recently, although a few pioneering studies have explored the application of state-of-the-art LLMs in chemistry, including a GPT-4-driven autonomous chemical reasoning system [14], CaR [15], MolReGPT [16], and ChemCrow [17], they have not adequately retrained LLMs using chemical data. Since chemical symbols are often regarded as a specialized language, models trained on large-scale natural language datasets struggle to fully comprehend various chemical symbols. More details about related works can be found in Section S2.

In this work, we consider the widely used SMILES (Simplified Molecular Input Line Entry System) [18] notation in chemistry and employ unified modeling to integrate it with natural language using LLMs. Following the modeling, we design multiple instruction tuning tasks and convert various open-source and our closed-source datasets into a large-scale instruction tuning dataset using prompt templates. Building upon our team’s self-developed BatGPT-15B [19], we expand its vocabulary with additional chemical terms and retrain it through instruction tuning, culminating in the model of BatGPT-Chem, a foundation large model for chemical engineering. Our model significantly surpasses existing large-scale chemical language models, such as ChemLLM [20] and ChemDFM [21], in terms of parameter amount, training data volume, and bilingual (English and Chinese) support. Therefore, it demonstrates substantially stronger performance across a wide range of chemical tasks.

Experimental results validate that BatGPT-Chem successfully incorporates chemical knowledge into retrosynthesis prediction, performs well under zero-shot conditions, and explicitly predicts reaction conditions in an end-to-end manner. These extensive capabilities set new benchmarks for the application of LLMs in the field of chemical engineering. In addition, we compare BatGPT-Chem with existing large models in the chemical field across tasks such as product inference, molecule design, molecule description, and yield prediction. As a general model, BatGPT-Chem also consistently performs better.

In summary, this study proposes a unified modeling framework for chemical and natural languages, enabling seamless integration of structured chemical representations and textual instructions. Based on this formulation, we develop a larger-scale, bilingual chemical foundation LLM called BatGPT-Chem with significantly more training data and stronger performance than prior methods and LLMs. BatGPT-Chem, trained on extensive chemical literature and symbolic data, offers enhanced accuracy and resilience in retrosynthesis analysis by interpreting relationships between textual descriptions and molecular structures. It can also predict reaction conditions beyond its training data. With unified modeling of natural language and SMILES, BatGPT-Chem also excels in tasks like molecule design, molecule description, product inference, and yield prediction, establishing itself as a versatile foundation model in chemistry and underscoring the potential of specialized instructional approaches in computational chemistry. Additionally, BatGPT-Chem’s deployment on an online server (https://compbio.sjtu.edu.cn/services/batgpt-chem/) offers chemists advanced synthesis suggestions, potentially accelerating innovation in drug manufacturing and materials science.

## Results and Discussion

### Retrosynthesis prediction

#### Retrosynthesis prediction benchmarks

Given a set of products, the objective is to generate precursors that synthesize the products. Similar to other LLMs, BatGPT-Chem has been trained on extensive data, making it challenging to estimate its zero-shot prediction ability for retrosynthesis prediction. To minimize overlap between test and training data and to explore a broader chemical space, we collect and organize 8 datasets with various reaction types to establish a new benchmark dataset for retrosynthesis prediction. We take reaction conditions (precursors) into account as much as possible. Details of the benchmark are provided in Table 1.

**Table 1.** The statistics of evaluation datasets

Dataset	# Products	# Reactions	With conditions
(a) SM	1	5,760	**✓**
(b) HTE BH	5	3,955	**✓**
(c) ELN BH	454	551	**✓**
(d) AAAA	189	273	**✓**
(e) Denmark	25	1,075	**✓**
(f) AHO	3,147	10,268	**✓**
(g) BioChem	16,838	33,687	**❌**
(h) USPTO-100	100	100	**❌**

SM, Suzuki–Miyaura; HTE BH, high-throughput experiments Buchwald–Hartwig; ELN BH, electronic laboratory notebook Buchwald–Hartwig; AAAA, asymmetric allylic alkylation with amine; Denmark, asymmetric *N*; *S*-acetal formation using CPA catalysts; AHO, asymmetric hydrogenation of olefins; BioChem, metabolites and biochemical reactions; USPTO-100, part of ChemLLMBench with 100 products

• The Suzuki–Miyaura (SM) dataset [22] contains one product of the SM cross-coupling reactions. There are 5,760 reactions on the combinations of 15 couplings of electrophiles and nucleophiles, 12 ligands (with a blank one), 8 bases (with a blank one), and 4 solvents [23].

• The high-throughput experiments Buchwald–Hartwig (HTE BH) dataset [24] contains 5 products of the Pd-catalyzed Buchwald–Hartwig C–N cross-coupling reactions. There are 3,955 reactions on the combinations of 15 aryl halides, 4 ligands, 3 bases, and 23 additives.

• The electronic laboratory notebook Buchwald–Hartwig (ELN BH) dataset [25] contains 454 products of the Pd-catalyzed Buchwald–Hartwig C–N cross-coupling reactions. It has 551 reactions with a wider range of reaction space than the HTE BH dataset.
• The asymmetric allylic alkylation with amine (AAAA) dataset [26] contains 189 products of 273 reactions.
• The Denmark dataset [27] contains 25 products of the asymmetric *N*, *S*-acetal formation using chiral phosphoric acid (CPA) catalysts. There are 1,075 reactions on the combinations of 43 catalysts, 5 imines, and 5 thiols.
• The asymmetric hydrogenation of olefins (AHO) dataset [28] contains 3,147 products. There are 10,268 reactions with 1,686 transition metal catalysts and 2,754 olefin substrates.
• The metabolites and biochemical reactions (BioChem) dataset [29] comprises 16,838 products specifically curated for the biosynthetic planning of natural products, including a total of 33,687 reactions. It does not contain information about reaction conditions.
• The USPTO-100 dataset is part of ChemLLMBench [30], containing 100 products (one product for one reaction) randomly sampled from the respective test sets. It does not contain information about reaction conditions.

We carefully examine the overlap between the pretraining dataset and the retrosynthesis benchmark. Except for the USPTO-100 dataset, which is a subset of the USPTO dataset with all 100 reactions included in the pretraining dataset, the other datasets have little or no overlap with the pretraining dataset. Without considering reaction conditions, the ELN BH, AHO, and BioChem datasets contain 3, 77, and 172 reactions from the pretraining dataset, respectively. Considering the reaction conditions, only the BioChem dataset has 53 overlapping reactions with the pretraining dataset. We also check the overlap within the retrosynthesis benchmark itself, finding that only the AAAA and AHO datasets share 1 and 8 reactions with the BioChem dataset, respectively. Given the minimal overlap among the datasets, we believe that it is sufficient to estimate the retrosynthesis prediction ability of models under zero-shot conditions without additional processing.

**Fig. 1.**
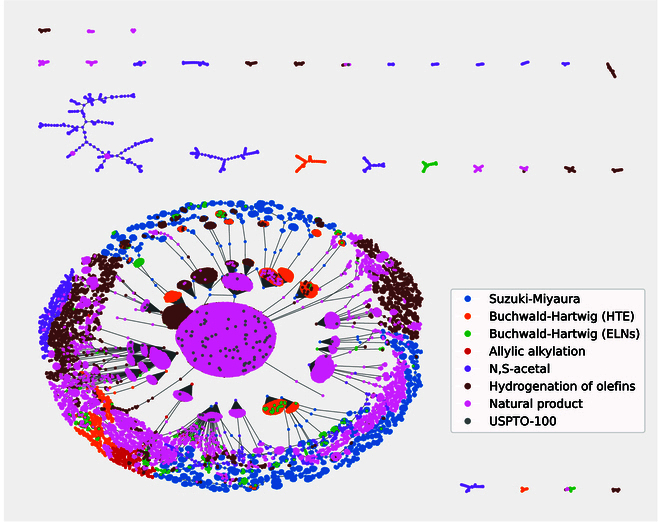
The annotated reaction graphs. The different fingerprints of reactions are visualized using a TMAP [32] algorithm.

Figure 1 presents annotated reaction graphs created using the DRFP [31] fingerprints, with colors corresponding to the 8 data sources. Despite slight fragmentation in the TMAP [32] sub-trees due to the large disparity in reaction numbers from different sources, related reaction types are well grouped. For example, in the lower left, the SM reactions are clustered continuously at the edges of the spherical tree, while the *N*, *S*-acetal reactions form disjointed sub-trees in the upper region. The various reaction types span a vast chemical reaction space, posing a significant challenge to the model’s generalization performance.

#### Evaluation metrics

Here, we introduce 2 new metrics, coverage of reactants (coverage) and intersection of reaction conditions (intersection), along with MaxFrag and Validity, to thoroughly evaluate retrosynthetic models. We also conduct multiple experiments by adjusting the Top-*k* hyperparameter of the model inference to evaluate its performance further. Note that the prediction is correct for products with multiple synthetic pathways as long as the model successfully predicts any of them.

Coverage of reactants indicates whether the model outputs include the true reactant molecules, thereby reflecting the accuracy of the reaction predictions. It differs from the coverage of precursors [33], which is related to whether the model can predict at least one valid precursor suggestion at the reaction level. Formally, we define it as

$${\text{Coverage}}_{\mathrm{Top}-k}=\frac{1}{\left|R\right|}\sum_{r\in R}I\left(\exists \;p\;\in \cup_{i=1}^{k}\;{\text{pred}}_{i}\left(r\right),\;s.t.p⊇\text{reactants}\left(r\right)\right),$$ (1)

where R denotes the reaction set, ${\text{pred}}_{i}\left(r\right)$ denotes the top-*i* prediction, and reactants(⋅) extracts reactants of a reaction.

Intersection of reaction conditions measures whether the model predicts any of the true reaction condition molecules. Directly predicting exact reaction conditions is extremely challenging, leading to common metrics such as Exact Match and Coverage being close to zero for most models. To address this, we propose the intersection accuracy metric, which relaxes the requirements and provides a more attainable measure of model performance when considering reaction conditions. Formally, we define it as

$$\begin{array}{c} \begin{array}{c} \begin{array}{c} \begin{array}{c} {\text{Intersection}}_{\mathrm{Top}-k}=\\ \frac{1}{\left|R\right|}\sum_{r\in R}I\left(\exists \;p\;\in \cup_{i=1}^{k}\;{\text{pred}}_{i}\left(r\right),\;s.t.\text{catalysts}\left(p\right)\cap \text{catalysts}\left(r\right)\neq \emptyset \right), \end{array} \end{array} \end{array} \end{array}$$ (2)

where catalysts(⋅) extracts catalysts of a reaction.

MaxFrag [34] assesses the model’s ability to identify the principal transformations in classical retrosynthesis. Prediction of the largest fragment focusing only on main compound transformations is the minimal information required for an efficient retrosynthesis route. Formally, we define it as

$$\begin{array}{c} {\text{MaxFrag}}_{\mathrm{Top}-k}=\\ \frac{1}{\left|R\right|}\sum_{r\in R}I\left(\exists \;p\;\in \cup_{i=1}^{k}\;{\text{pred}}_{i}\left(r\right),\;s.t.\text{maxfrag}\left(p\right)=\text{maxfrag}\left(r\right)\right), \end{array}$$ (3)

where maxfrag(⋅) extracts the max fragment of a reaction.

Validity [35] measures the proportion of SMILES codes predicted by the model that are chemically valid and do not violate chemical principles. Formally, we define it as

$${\text{Validity}}_{\mathrm{Top}-k}=\frac{1}{k\left|R\right|}\sum_{r\in R}\sum_{i=1}^{k}I\left(\text{valid}\left({\text{pred}}_{i}\left(r\right)\right)\right),$$ (4)

where valid(⋅) detects whether the given SMILES of a reaction is valid.

#### Baselines

We compare our method against a diverse set of strong baselines, including both domain-specific and general-purpose LLMs, as well as classical learning-based models. All LLMs are evaluated using the efficient inference engine vLLM [36] v0.5.4 with beam search, a maximum token length of 512, and top-10 sampling. To ensure robustness, we use 5 random seeds, and all other settings follow the default configuration of vLLM. An ablation study for BatGPT-Chem is provided in Table S1. The baseline LLMs and traditional methods are listed below:

Llama-3-8B [37] is one of the most powerful open-source English models currently available, outperforming previous general-purpose models across multiple interdisciplinary domains such as biology, chemistry, and physics.

Falcon-40B [38] series is also one of the most powerful open-source English models, with strong performance in STEM (science, technology, engineering, and mathematics) fields. We use the Falcon-40B version for our experiments.

ChemDFM-13B [21] is a large chemistry model with strong capabilities in retrosynthesis prediction, molecule design, molecule description, and so on. It is further trained on chemical data based on Llama2-13B and supports only English.

ChemLLM-20B [20] is another large chemistry model trained on InternLM2 [39], primarily designed for expert-level conversations in the field of chemistry. We use the ChemLLM-20B-Chat-DPO version of the model for our experiments.

LLaMA-4-Scout-17B [40] is an instruction-tuned model from the LLaMA-4 family, designed for improved reasoning and factual accuracy. Although it is not chemistry-specific, its strong general capabilities make it a competitive baseline in our setting.

Qwen-3-32B [41] is a large-scale general-purpose model with enhanced multilingual and reasoning abilities. Despite being a general LLM, Qwen-3-32B demonstrates strong potential in domain adaptation and is included to evaluate the upper bounds of general model transferability.

GPT-4 [42] is the commercial model released by OpenAI. While its architecture and training details are undisclosed, it achieves state-of-the-art performance on a wide range of tasks, including chemistry-related question–answer (QA) and reasoning problems.

UAGNN [43] is a graph neural network-based model that uses atom-level and bond-level information for reaction outcome prediction.

Chemformer [44] is a Transformer-based model trained on SMILES representations for tasks such as retrosynthesis prediction.

#### BatGPT-Chem achieves exceptional accuracy in identifying reactants

**Fig. 2.**
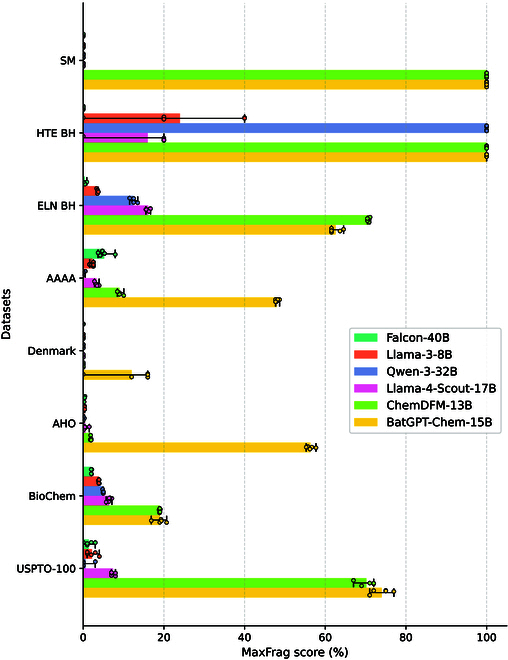
Top-10 MaxFrag scores for zero-shot retrosynthesis prediction benchmark. Results are averaged over 5 random runs with min–max error bars.

In the MaxFrag score analysis presented in Fig. 2, BatGPT-Chem achieves state-of-the-art performance across most datasets, except for the ELN BH dataset, where it is surpassed by ChemDFM-13B. Since we use various prompts with ChemLLM-20B and consistently obtain a score of 0, we exclude this model from Fig. 2. General-purpose LLMs (Falcon-40B, Llama-3-8B, Qwen-3-32B, and Llama-4-Scout-17B) display significantly weaker performance against chemistry-specific LLMs across all datasets, where only Qwen-3-32B obtains a great 100% score on the HTE BH dataset. For the complex reaction class of asymmetric *N*, *S*-acetal formation from the Denmark dataset, other models fail to identify the largest fragments in any of the 25 reactions, while BatGPT-Chem successfully predicts key fragments in 4 reactions. To provide a clearer demonstration of BatGPT-Chem’s retrosynthesis prediction capabilities, we present several examples in Figs. S1 to S7.

Notably, in reactions from the AHO dataset, which are relatively simple and primarily involve the addition of hydrogens to reactants to obtain the product, other models exhibit extremely poor performance. In contrast, BatGPT-Chem maintains a high accuracy rate. Among the 10,268 reactions in the AHO dataset, it achieves an accuracy of 56.2%. This accuracy notably exceeds that of the second-best ChemDFM-13B by over 30 times.

**Fig. 3.**
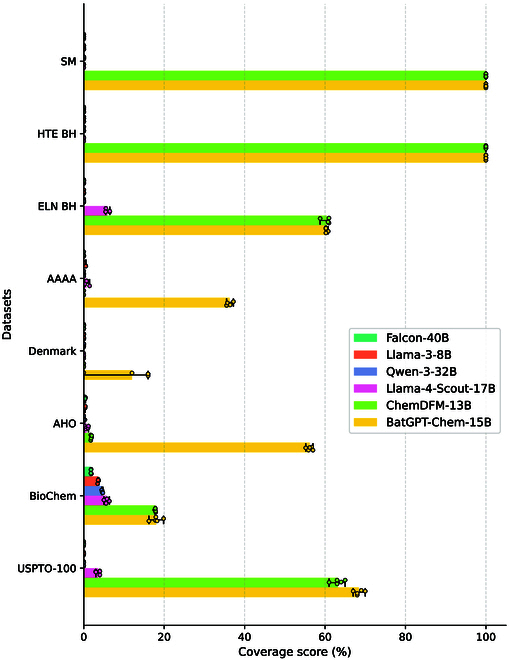
Top-10 coverage scores for zero-shot retrosynthesis prediction benchmark. Results are averaged over 5 random runs with min–max error bars.

In direct synthesis scenarios that require precise identification of all interacting components, we evaluate the predictive performance of models by assessing their ability to cover all reactants in a reaction, i.e., coverage. The results, presented in Fig. 3, align with trends seen in the MaxFrag metric, yet with notably lower coverage scores. For instance, BatGPT-Chem’s performance on the ELN BH and USPTO-100 datasets declines from 62.6% to 60.3% and 74.0% to 68.4%, respectively. Coverage score of ChemDFM-13B even drops to 0% on the SM and AAAA datasets. While classical retrosynthesis primarily targets key transformations, the ability to accurately predict additional reactants enhances the completeness of retrosynthetic analyses.

In this evaluation, we assess language models on reactant prediction using the MaxFrag and Coverage metrics instead of the traditional exact match Top-*n* accuracy. Many LLMs do not differentiate between reactants and reaction conditions during training, typically using the “.” notation to segregate molecules within reaction SMILES strings. This convention complicates the automatic extraction of condition components from reaction SMILES, making it difficult to discern reactants from the output strings, and thus potentially rendering the application of the exact match metric unfair. More results for retrosynthesis prediction can be found in Table S2.

#### BatGPT-Chem capably predicts reaction conditions explicitly

Beyond the identification of reaction reactants, the prediction of appropriate reaction conditions, like catalysts and solvents, remains a crucial and challenging subsequent task [33]. Due to the significant complexity involved in accurately and comprehensively predicting reaction conditions, LLMs often struggle to yield reasonable results when assessed using Exact Match and MaxFrag metrics. Therefore, here, we employ the “Intersection” metric, as described in Baselines, to more effectively evaluate the model’s capability in predicting reaction conditions.

**Fig. 4.**
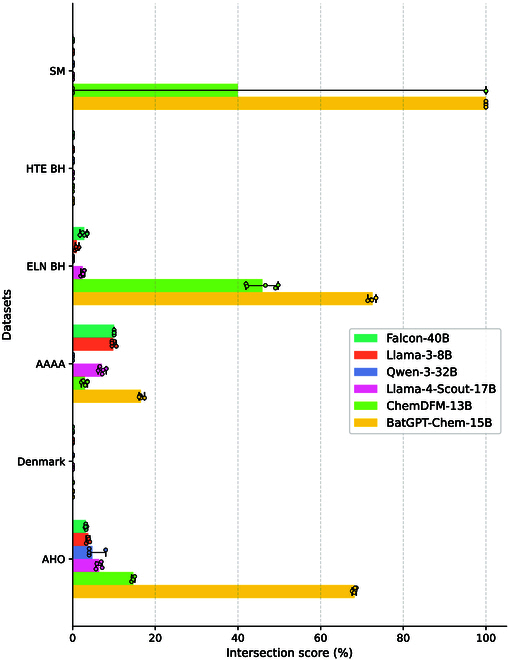
Top-10 intersection scores for zero-shot retrosynthesis prediction benchmark. Results are averaged over 5 random runs with min–max error bars. Note that for the HTE BH and Denmark datasets, all models fail to predict correct conditions and get scores of 0.

We assess the accuracy of reaction condition predictions across 6 datasets that contain reaction condition information. As shown in Fig. 4, BatGPT-Chem outperforms other methods by large margins in terms of intersection rate.

This demonstrates BatGPT-Chem’s exceptional ability to predict reaction conditions and complete retrosynthesis routes effectively. However, it is important to note that on the HTE BH and Denmark datasets, all models consistently fail to present any feasible reaction conditions, highlighting the inherent challenges of this task.

Moreover, as mentioned in BatGPT-Chem capably predicts reaction conditions explicitly, while many methods do not distinguish between reactants and reaction conditions within their reaction strings, BatGPT-Chem’s training corpus separates these 2 elements with a “>”. This distinction enables BatGPT-Chem to explicitly predict reaction conditions through prompting, markedly enhancing its capacity to provide comprehensive retrosynthesis routes.

**Fig. 5.**
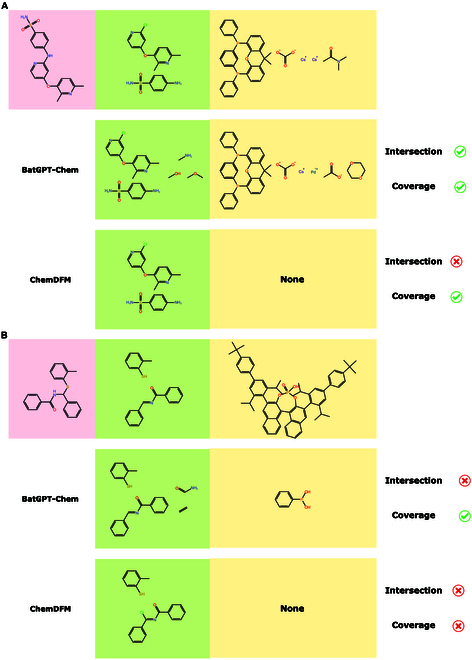
Comparison of predictions between BatGPT-Chem and ChemDFM-13B where products are displayed in pink blocks, reactants are in green blocks, and reaction conditions are in yellow blocks. (A) Example from the ELN BH dataset. (B) Example from the Denmark dataset.

To further illustrate the predictive capabilities concerning reaction conditions, we analyze 2 specific cases: one from the ELN BH dataset and another from the Denmark dataset, comparing BatGPT-Chem with the top-performing baseline, ChemDFM-13B. As shown in Fig. 5A, BatGPT-Chem successfully predicts most conditions, including the catalyst and metal, only missing one reaction condition. In contrast, ChemDFM-13B fails to generate any correct conditions. In this reaction, the catalyst’s structure is relatively complex, and multiple reaction conditions are required for this retrosynthesis pathway. In cases where reaction conditions are exceedingly complex, it becomes even more challenging for models to make accurate predictions. As can be seen in Fig. 5B, BatGPT-Chem covers all reactants but fails to predict the catalyst, whereas ChemDFM-13B correctly identifies only one reactant. Note that BatGPT-Chem occasionally recommends supplementary simple small molecules among the reactants, which have a negligible influence on the determination of the reaction pathway.

#### BatGPT-Chem excels in generating multiple viable retrosynthesis routes

**Fig. 6.**
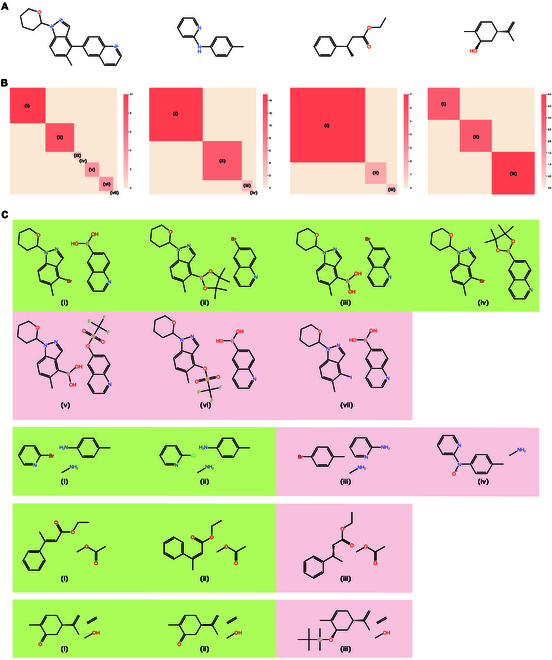
Analysis of predictions generated by BatGPT-Chem. (A) Products sampled from the SM, HTE BH, AHO, and BioChem datasets. (B) Numbers of prediction within Top-*k*. (C) Details of predictions where green means that ground truth is covered and red means not.

After achieving accurate predictions for reactants and reaction conditions, the focus shifts to evaluating the model’s ability to generate diverse, correct, and even novel predictions. Due to the stochastic nature of LLMs, retrosynthesis predictions sampled from BatGPT-Chem for a fixed product will not be unique. To assess BatGPT-Chem’s capability to propose multiple retrosynthesis routes, we examine 4 representative products (Fig. 6A) from the benchmark. We sample the Top-30 predictions for the SM and HTE BH datasets and the Top-10 predictions for the AHO and BioChem datasets. Figure 6B and C displays the frequencies and detailed predictions, showcasing BatGPT-Chem’s capacity to provide diverse retrosynthesis routes.

Specifically, for the product from the SM dataset, BatGPT-Chem generates 7 different predictions, 4 of which (i to iv) match the ground truths with a frequency ratio of 23/30. The remaining predictions are not in the dataset but still contain valid functional groups. For the product from the HTE BH dataset, BatGPT-Chem provides 4 different predictions, 2 of which (i and ii) match the ground truths with a frequency ratio of 26/30. Notably, prediction iii, not included in the original dataset, is found in Reaxys [45,46] (ID: 39015457), highlighting the model’s capability to predict reactions outside the benchmark dataset.

For the product from the AHO dataset, BatGPT-Chem produces 3 different predictions, 2 of which (i and ii) match the ground truths with a frequency ratio of 9/10. For this chiral product, BatGPT-Chem successfully predicts all cis-trans isomerism (i and ii) and even provides the SMILES without cis-trans information (iii). For the product from the BioChem dataset, BatGPT-Chem makes 4 different predictions, 2 of which (i and ii) match the ground truths with a frequency ratio of 6/10. This is also a chiral product, and BatGPT-Chem accurately predicts different chiral configurations *S* (i) and *R* (ii). Again, BatGPT-Chem gives some extra simple small molecules in the reactants for some predictions.

These results emphasize BatGPT-Chem’s ability to capture the major backbone of the reactant molecules, provide diverse predictions for retrosynthesis pathways, and predict reactions beyond the benchmark dataset, further demonstrating its robustness and versatility in chemical synthesis planning.

In retrosynthesis, the feasibility of synthesizing a single product from various precursors adds complexity to the evaluation of model predictions. For example, in the reaction R − R1 + NH3 → R − NH2, multiple substituents for R1, such as -OH, -Cl, -Br, -I, or -F, are valid, each leading to correct predictions [34]. The primary distinction among these options stems from their respective reaction rates and yields.

While the “MaxFrag” metric does not fully address this issue, it still represents an effort to better manage such data ambiguities during the validation process. Instead of devising a new metric, our approach focuses on addressing this challenge from a data-centric perspective to enhance the comprehensiveness of performance evaluation. Specifically, during dataset compilation for model evaluation, we endeavor to gather as many synthetic pathways for the same product as feasible. Subsequently, during the assessment phase, a prediction is deemed successful if it aligns with any of the collected retrosynthesis pathways for that product.

#### BatGPT-Chem generates outputs with high validity

**Fig. 7.**
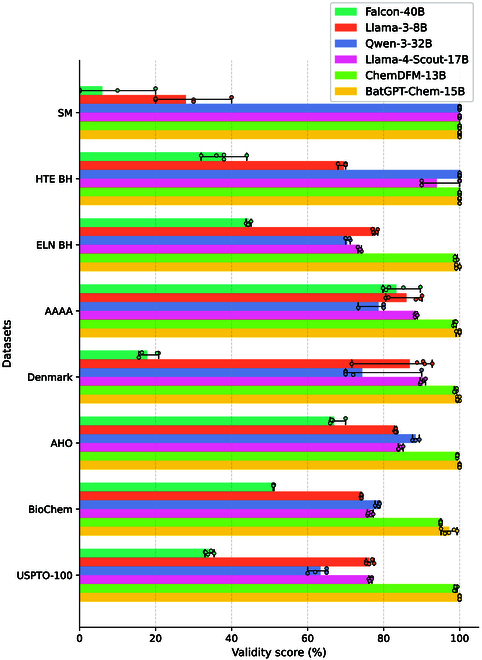
Top-10 validity scores for zero-shot retrosynthesis prediction benchmark. Results are averaged over 5 random runs with min–max error bars.

As LLMs sometimes produce SMILES representations of molecules that may not be valid or chemically plausible, we employ RDKit [47] v2024.3.5 to verify the validity of molecules generated by these models. BatGPT-Chem consistently achieves high validity rates as presented in Fig. 7, nearing or reaching 100% across all datasets, thereby confirming its strong grasp of the chemical language. Given that chemical symbols can be considered a specialized language domain, models primarily trained on general natural language corpora often fail to fully comprehend them. BatGPT-Chem can avoid tedious post-processing of grammatical corrections [48] to fix the syntax errors of outputs. Furthermore, it successfully interprets the cis-trans and chiral information inherent in chemical language, as illustrated in Fig. 6C.

### Exploration of broader capabilities

**Fig. 8.**
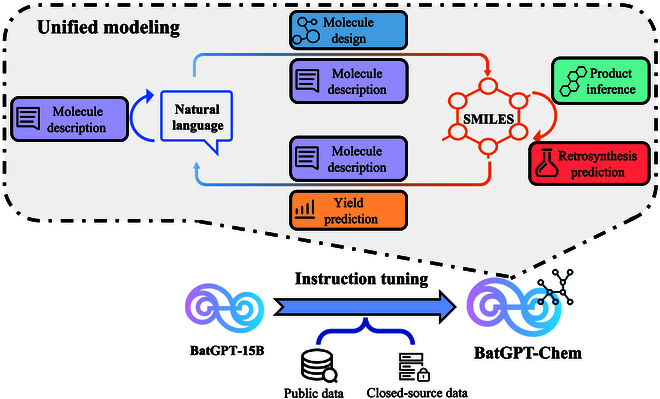
An illustration of unified modeling between natural language and SMILES. We use our team’s self-developed BatGPT-15B as the base model. Following the unified modeling, BatGPT-Chem is derived from BatGPT-15B through instruction tuning on both public data and closed-source data. It is capable of performing chemical tasks involving arbitrary conversions between natural language and SMILES.

By treating chemical notation as a specialized language, we leverage LLMs for unified modeling across various conversions: natural language to SMILES, SMILES to natural language, SMILES to SMILES, and natural language to natural language. This approach enables chemistry tasks such as molecule description, molecule design, product inference, and retrosynthesis prediction. Our modeling approach, illustrated in Fig. 8, includes bidirectional conversions for molecule description, natural language to SMILES for molecule design, and SMILES to SMILES for product inference and retrosynthesis. We also incorporate a task for yield prediction. We present detailed prompt templates and prompt examples in Sections S5 and S6.

Therefore, this section evaluates BatGPT-Chem’s versatility in chemistry by comparing its performance with other models on tasks other than retrosynthesis, including molecule design, molecule description, product inference, and yield prediction. Specifically, BatGPT-Chem, ChemDFM-13B, and ChemLLM-20B are tested using identical prompts, allowing for a direct comparison of their outputs. While these tasks have specific benchmarks that typically favor specialized models and traditional machine learning approaches, BatGPT-Chem stands out as a general-purpose large model for chemistry. Despite the challenge of outperforming task-specific models on individual tasks, this section highlights the advantages of BatGPT-Chem over baseline large models through several zero-shot prompt case studies.

#### Molecule design

BatGPT-Chem can achieve molecule design when predicting reactants, catalysts, or products by imposing conditions that the predicted molecules should satisfy. These conditions come from RDKit’s MoleculeDescriptors module, such as average molecular weight, exact molecular weight, and the number of nitrogens and oxygens.

**Fig. 9.**
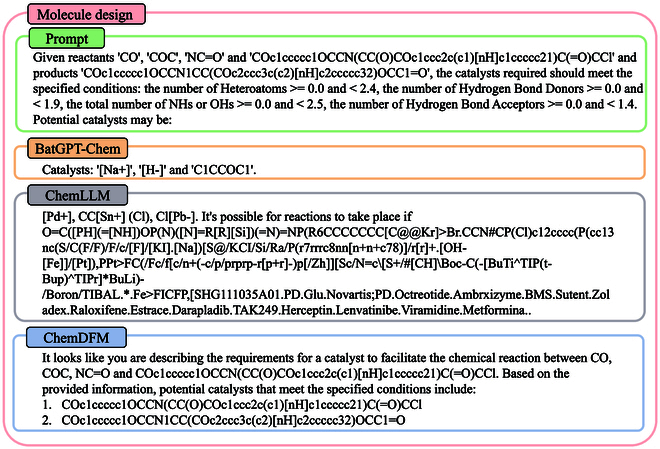
A case of molecule design involves providing the reactants and products of a given reaction and asking the model to predict a suitable catalyst, with only the prediction from BatGPT-Chem meeting the criteria.

As shown in Fig. 9, we demonstrate that for a given reactant and product in a reaction, an LLM is required to predict a catalyst that meets the specified conditions. Among the predictions, only the catalyst “[Na+].[H-].C1CCOC1” predicted by BatGPT-Chem meets the conditions. The prediction from ChemDFM-13B, “[Pd+]Cl[Pb-]CC[Sn+](Cl)”, has 5 heteroatoms, which does not satisfy the condition of being between 0.0 and 2.4. The 2 predictions from ChemDFM-13B have 7 and 8 heteroatoms, respectively, which also do not meet the criteria; their hydrogen bond acceptors are both 5, failing to satisfy the condition of being between 0.0 and 1.4. We conduct multiple tests, and the ratio of catalysts predicted by BatGPT-Chem that meet the criteria exceeds 90%, while the other 2 models are below 10%.

#### Molecule description

BatGPT-Chem supports bidirectional molecule descriptions, where a description of a molecule can be provided, and the model will output the possible SMILES code and corresponding IUPAC (International Union of Pure and Applied Chemistry) name. Alternatively, a SMILES code or IUPAC name can be given, and the model will generate the corresponding molecule description.

**Fig. 10.**
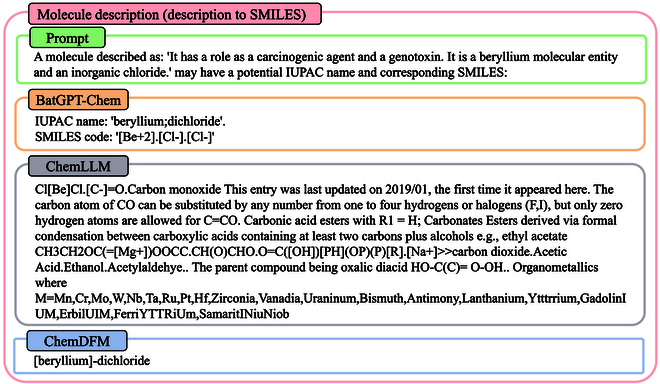
A case of converting a molecule description to SMILES and IUPAC names. Both BatGPT-Chem and ChemDFM-13B generate the correct IUPAC name, with BatGPT-Chem also providing the corresponding SMILES. However, ChemLLM-20B fails to produce the correct IUPAC name or SMILES.

Description to SMILES: In Fig. 10, we present an example where a molecule description is input, and the models output the corresponding IUPAC name and SMILES. Both BatGPT-Chem and ChemDFM-13B output the correct IUPAC name “beryllium;dichloride”. However, BatGPT-Chem also follows the instruction and provides the corresponding SMILES, while ChemDFM-13B does not. ChemLLM-20B, on the other hand, does not output the correct IUPAC name or SMILES.

**Fig. 11.**
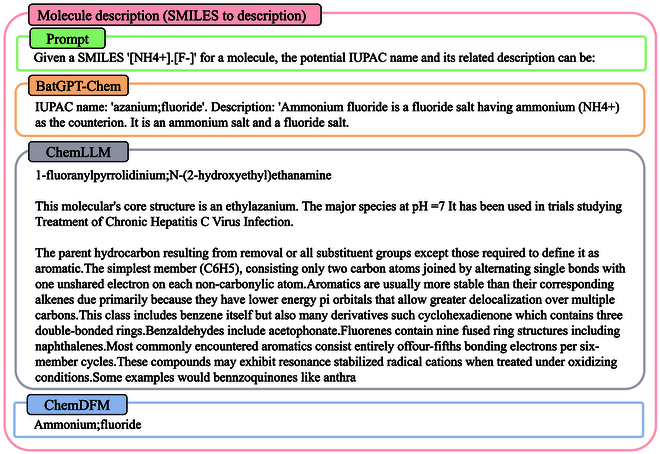
This case demonstrates the conversion of a SMILES code to a molecule description and IUPAC name. Both BatGPT-Chem and ChemDFM-13B generate the correct IUPAC name, but only BatGPT-Chem provides the corresponding molecule description. In contrast, ChemLLM-20B fails to produce the correct IUPAC name or description.

SMILES to description: In Fig. 11, we present a case where a given SMILES code is provided, and the models generate the corresponding IUPAC name and molecule description. Similarly, BatGPT-Chem and ChemDFM-13B output the correct IUPAC name, but only BatGPT-Chem follows the instruction and provides the corresponding description, while ChemDFM-13B does not. ChemLLM-20B, on the other hand, outputs completely incorrect names and descriptions.

#### Product inference

**Fig. 12.**
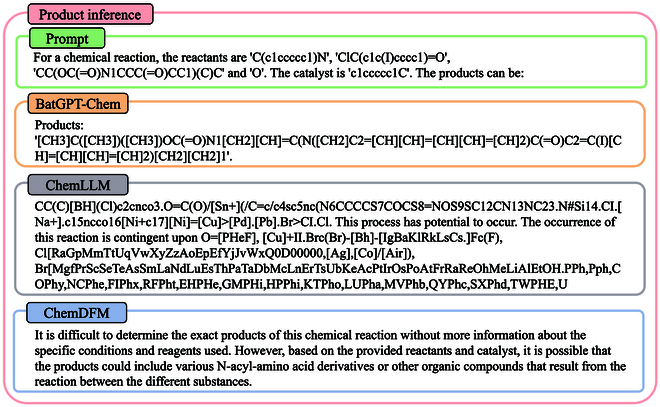
A case of product inference, where only BatGPT-Chem successfully and accurately predicts the product given the reactants and catalyst of a specified reaction equation.

We present an example of product inference in Fig. 12, where we provide the reactants and catalysts of a given reaction and ask the model to predict the corresponding product. Only BatGPT-Chem predicts the ground truth, while ChemLLM-20B first outputs an unrelated reaction equation and then produces some gibberish. ChemDFM-13B indicates that it lacks sufficient information to make a prediction. Overall, BatGPT-Chem demonstrates the best prediction performance.

#### Yield prediction

**Fig. 13.**
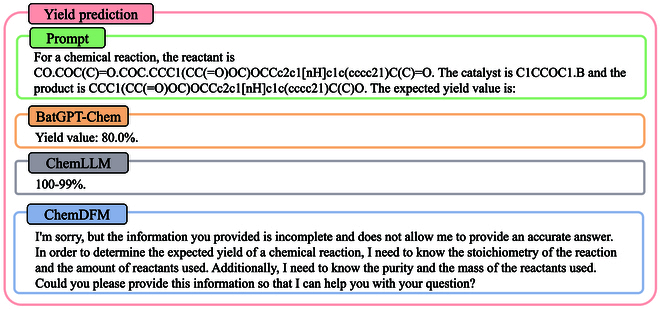
A case of yield prediction, where BatGPT-Chem provides a correctly formatted yield prediction, ChemLLM-20B gives a somewhat misleading result that may indicate between 99% and 100%, and ChemDFM-13B states that the information is insufficient and does not provide a yield prediction.

Yield prediction involves providing the reactants, catalysts, and products of a given chemical reaction and asking the model to predict the possible yield of that reaction. We present an example of yield prediction in Fig. 13, where the chemical reaction is given and the model is asked to predict the corresponding yield. The ground truth yield for this reaction from the dataset is 98%.

In this case, although the yield value predicted by BatGPT-Chem deviates from the ground truth, the output format is generally correct and presents the yield prediction clearly. In contrast, ChemLLM-20B outputs a yield of “100-99%”, which can be misleading; we suspect that this reflects a predicted yield between 99% and 100%. Notably, many yields in the current training data are missing, and the same reaction may correspond to multiple yield values in the dataset. As a result, the quality of the existing yield prediction dataset is relatively low, which could contribute to the inaccuracies in our model’s yield predictions. We plan to collect more high-quality yield prediction data in the future to enhance BatGPT-Chem’s capabilities in this area. Additionally, ChemDFM-13B states that the provided information is insufficient to predict the yield. However, the existing training datasets do provide the reactants, catalysts, products, and yield for a given reaction, indicating that this information should be adequate for making predictions. More results for yield prediction can be found in Table S3.

### Different representation for molecules

While the SMILES notation is widely used for molecular representation in computational chemistry, it has limitations such as syntactic ambiguity and non-unique encodings for certain molecular structures [49]. Alternative formats like SELFIES [50], DeepSMILES [49], and graph-based representations have been proposed to overcome these challenges. However, our decision to adopt SMILES as the primary representation in BatGPT-Chem is based on the following key considerations:

• Ubiquity: SMILES remains the most widely used representation in computational chemistry [51] and is deeply embedded in major chemical databases (e.g., USPTO and PubChem) and toolkits (e.g., RDKit), allowing for seamless integration with existing infrastructures.
• Sequential compatibility: As a string-based format, SMILES aligns naturally with the sequential processing paradigm of LLMs, enabling unified modeling with natural language.
• Rapid convertibility: SMILES can be efficiently converted to other molecular representations (e.g., SELFIES and InChI) through established algorithms. This enables BatGPT-Chem to achieve cross-representation adaptability with minimal additional training, either through limited fine-tuning data or simply by incorporating conversion tools at model interfaces, thus extending its capability to process alternative formats like SELFIES [52] without architectural modifications.

Actually, our work does not limit itself to SMILES alone. Representations such as IUPAC names are also incorporated during model training to enhance chemical language understanding. Future work will explore integrating SELFIES to enhance robustness against invalid outputs and investigate graph-based transformers for explicit stereochemical modeling. Nevertheless, SMILES’ dominance in legacy datasets and its alignment with LLM architectures make it an indispensable foundation for current large-scale chemical models.

### Safety filtering during training

To minimize the risk of misuse, we implement a rigorous safety filtering pipeline. We collect as many hazardous molecules as possible from publicly available Material Safety Data Sheets (MSDS) and controlled substances from the drug catalog issued by the People’s Republic of China. These molecules, known to be toxic, harmful, addictive, or explosive, are identified via exact SMILES matching and removed from the training set.

Additionally, we perform keyword-based filtering on molecular annotations and descriptions using terms related to toxicity, harmfulness, addiction, or explosive potential. This process flags and removes 0.0002% of entries.

### Post-training risk assessment and mitigation

After training, we conduct adversarial prompting with 500 high-risk queries to evaluate whether BatGPT-Chem might generate harmful compounds. No known toxic, addictive, or explosive molecules are observed among the outputs. For example, when prompted with “It has a role as a toxic and addictive drug”, the model outputs glycerine, a harmless compound, demonstrating its resistance to dangerous generation.

Looking forward, we plan to implement real-time prompt filtering and SMILES-based output screening in the trial platform to prevent dual-use misuse. We also support community engagement and responsible release practices, including transparency documentation and collaboration with chemistry and AI safety communities to strengthen safeguards.

## Conclusion

LLMs have made substantial progress across various fields, demonstrating significant potential to spearhead advancements in AI for Science. Notably, their proficiency in processing sequential data makes them particularly apt for the chemical domain, where common representations such as SMILES also adopt a sequential format. Considering the natural potential of LLMs to learn and predict chemical structures and reactions, we develop BatGPT-Chem, a pioneering large-scale model tailored for chemical engineering, to address 3 critical limitations prevalent in existing AI models: (a) a deficit in comprehensive molecular and chemical reaction knowledge; (b) oversight of reaction conditions; and (c) inadequate generalization across diverse chemical reactions.

BatGPT-Chem distinguishes itself by its extensive training corpus, which covers a wide range of chemical literature and chemical string data, i.e., SMILES strings. The implementation of carefully crafted prompt templates and tailored instruction-tuning data during pretraining has significantly enhanced its capacity to decipher both natural and chemical languages. This advancement is reflected in its improved accuracy in predicting reactants and the near-perfect validity of its output. Particularly noteworthy is BatGPT-Chem’s performance on comprehensive benchmark datasets, demonstrating remarkable zero-shot retrosynthesis prediction capacities that hold practical implications for real-world applications.

A unique aspect of BatGPT-Chem compared to other LLMs is its explicit handling of reaction conditions. By directly extracting reaction conditions from datasets and creating specific prompts to predict them, BatGPT-Chem shows superior ability to elucidate components such as solvents and catalysts.

When predicting reaction conditions, nongenerative models can only deal with a fixed set of molecules and are usually modeled as multi-classification or multi-label problems [53–56], which greatly limits the generalization ability of these models. These methods use a post-processing approach in retrosynthesis, where reaction conditions are predicted after the reactants and products are known. This 2-stage processing requires additional training of the model, is not simple enough to use, and makes it more difficult to ensure the stability of the model. Contrastingly, generative models like BatGPT-Chem can predict a wide variety of reaction condition molecules, are not constrained by a finite set, and can offer novel and heuristic predictions in an end-to-end manner. Since most of the reaction condition information is stored using raw text [57], constructing datasets for machine learning is inherently time-consuming and laborious, including steps such as extracting text, removing errors, and converting to computer-readable sequences [23,56]. BatGPT-Chem can provide reference reaction conditions for retrosynthesis prediction datasets like BioChem [29], which do not contain information on reaction conditions. This capability can help build more comprehensive and enriched datasets of chemical reactions, facilitating the use of machine learning in chemical reaction modeling.

Moreover, BatGPT-Chem excels in generating diverse and viable retrosynthesis pathways, providing valuable insights for chemists. In fact, the cases where multiple paths correspond to the same product are rare in the pretraining datasets; thus, BatGPT-Chem’s ability to suggest various feasible routes can be attributed to its profound understanding of chemical reaction mechanisms. By optimizing beam search strategies and temperature settings, the model adeptly balances diversity and correctness. In contrast, efforts to enhance output diversity in other LLMs through elevated temperature settings often result in an increased error rate.

In conclusion, BatGPT-Chem sets new benchmarks for effective and dependable AI-driven retrosynthesis prediction. Furthermore, by unifying natural language and SMILES notation, BatGPT-Chem excels in tackling diverse chemical challenges, outperforming existing chemical LLMs across multiple tasks, and elevating both efficiency and innovation. However, the work is still tempered by the quality of the data, primarily from open-access sources, with high-quality model description data often confined to proprietary databases. Despite endeavors to enrich reaction condition data and compile comprehensive retrosynthesis pathways, gaps remain. Future improvements will likely require collective efforts from across the scientific community. Another constraint is the scope of chemical languages covered; currently focused on SMILES, exploring additional string-based representations like SELFIES [50] could broaden our model’s utility, paving the way for its application to a greater spectrum of chemical reactions.

## Materials and Methods

### Base model

We select our team’s self-developed BatGPT-15B model [19] as the base model for instruction tuning. BatGPT-15B is a large bilingual model for both Chinese and English, which adopts the Llama architecture and utilizes Multi-Query Attention technology [58], supporting an input length of 32,000.

It innovatively adopts a bidirectional autoregressive strategy during the pretraining phase. In addition to the traditional autoregressive training, where the model predicts the next token based on the previous one, a reverse autoregressive strategy is also employed, training the model to predict the previous token based on the next one. This approach helps mitigate the model’s hallucination issue. BatGPT has demonstrated excellent performance in STEM tasks on public benchmarks, such as CMMLU [59], making it highly suitable for AI4Science applications, such as in the field of chemistry.

### Chemistry tasks and prompt templates

Following the modeling approach outlined above, we focus on the key tasks in the chemistry domain: molecule description, Molecule design, product inference, retrosynthesis prediction, and yield prediction. We construct instruction tuning datasets based on existing chemical, drug, and medicine datasets using prompt templates to train models capable of addressing these tasks.

Retrosynthesis prediction. Retrosynthesis prediction is a crucial task for chemistry. It involves inferring possible reaction pathways and conditions by given product molecules, thereby reverse-predicting the synthetic route to generate the product. Retrosynthesis prediction enables researchers to explore and discover new organic molecular structures more rapidly, which is essential for organic synthesis and drug discovery. We train the model’s retrosynthesis prediction capability using 2 subtasks: (a) Reactant and catalyst prediction: Given a product, predict the potential catalysts and reactants that may be required. (b) Reactant prediction: Given a product and catalysts, predict the reactants.

Product inference. Product inference aims at predicting the products based on given starting materials and specific reaction conditions, which holds significant importance in fields such as organic synthesis and drug design. We train the model’s product inference capability using 2 subtasks: (a) Product and catalyst prediction: Given reactants, predict the potential catalysts and products that may be involved. (b) Product prediction: Given products and catalysts, predict the reactants involved.

Molecule design. Molecule design is a field involving the creation of new molecules using theoretical and computational methods to produce molecular structures with specific properties or functionalities. This field plays a crucial role in various domains, including chemistry, drug design, and materials science. Molecule design aims to systematically generate molecules with desired properties and activities to meet specific application needs. This work fully considers over a hundred molecular properties, such as molecular weight, valence electron count, Balaban J value, BertzCT value, number of heavy atoms, number of NHs or OHs, and number of nitrogen and oxygen atoms. It is hoped that the LLM can take into account researchers’ specific requirements for molecular properties of catalysis, products, and reactants of chemical reactions. To train the model’s molecule design capability, the following 3 tasks are adopted: (a) Specifying catalyst molecular properties: Given reactants to produce a specific product, the catalyst is required to meet certain properties. (b) Specifying reactant and catalyst molecular properties: Given the desired product to be synthesized, both reactants and catalysts are required to meet certain properties. (c) Specifying reactant, catalyst, and product properties: The model is required to provide a chemical reaction with specified molecular properties for reactants, catalysts, and products.

Molecule description. Molecule description refers to using computational models to predict and describe the function, effects, and related properties of a molecule given its name, SMILES, or other representations. We adopt the following 8 subtasks to train the model’s molecule description capability. We not only utilize chemical data for training to enable the model to fully understand and perceive the correspondence between molecular names in both English and Chinese, molecule descriptions, molecule SMILES, and molecule IUPAC names, thus obtaining strong molecular description capabilities, but also incorporate some pharmaceutical data to enhance the model’s ability in the pharmaceutical field: (a) Given the molecular Chinese name, generate the English name and description. (b) Given the molecular English name, generate the Chinese name and description. (c) Given the molecular description, generate the Chinese name and English name of the molecule. (d) Given the molecular description, generate the IUPAC name and SMILES code of the molecule. (e) Given the molecular SMILES code, generate the IUPAC name and SMILES code of the molecule. (f) Given the molecular IUPAC name, generate the SMILES code and description of the molecule. (g) Given the Chinese name of a drug, generate the English name and description. (h) Given the English name of a drug, generate the Chinese name and description. (i) Given the description of a drug, generate the Chinese name and English name.

Yield prediction. Yield prediction in chemical reactions refers to the estimation, through experimental or computational methods, of the ratio between the actual quantity of products generated in a chemical reaction and the theoretically maximum possible yield. We also train the model by predicting corresponding yields for given chemical reactions.

### Data source

We utilize publicly available high-quality datasets in the field of chemistry, as well as closed-source datasets within our team, as the raw datasets. Then, we process the source dataset into instruction tuning datasets. The number of entries in each source dataset is shown in Table 2.

**Table 2.** Number of entries in source dataset

Source	Dataset	Number of entries
Open-source	USPTO	2,746,598
	CHEBI	3,978,879
	CJHIF	2,962,940
	PubChem	1,378,095
	Text2Mol	12,951
Closed-source	Drug Instruction	37,288
	Organic Compound Manual	59,771
	Formula and Name Reference Table	62,967
	SMILES, IUPAC and Descriptions Reference Table	45,770

#### Publicly available datasets

• USPTO [60]: USPTO collects reaction data extracted through text mining from United States patents published between 1976 and September 2016.

• CHEBI [61]: Chemical Entities of Biological Interest (CHEBI) is a freely available dictionary of molecular entities focused on “small” chemical compounds. The term “molecular entity” refers to any constitutionally or isotopically distinct atom, molecule, ion, ion pair, radical, radical ion, complex, conformer, etc., identifiable as a separately distinguishable entity. The molecular entities in question are either products of nature or synthetic products used to intervene in the processes of living organisms.

• CJHIF [57]: Chemical Journals with High Impact Factors (CJHIF) is a high-quality dataset containing a large number of chemical reaction equations extracted from various chemical journals.

• PubChem [62]: PubChem is an open chemistry database at the National Institutes of Health (NIH), which mostly contains not only small molecules but also larger molecules such as nucleotides, carbohydrates, lipids, peptides, and chemically modified macromolecules.

• Text2Mol [9]: Text2Mol provides a large amount of data containing natural language descriptions of molecules.

#### Closed-source datasets

• Drug Instruction: We collect a large number of drug names, drug descriptions, and corresponding molecular formulas from drug instructions to enhance the model’s capabilities in the pharmaceutical domain.

• Organic Compound Manual: We have a large collection of private organic compound manuals, containing information such as organic compound names, compound descriptions, and compound SMILES.

• Formula and Name Reference Table: We have collected a large amount of publicly available data on compound names and their corresponding molecular formulas.

• SMILES, IUPAC, and Descriptions Reference Table: We have collected data on SMILES, IUPAC names, and their corresponding molecular descriptions.

### Dataset preparation

In this section, we describe the dataset preparation process, including data cleaning, data augmentation, prompt construction for instruction tuning, duplicate removal, and safety filtering procedures.

#### Data cleaning

The publicly available datasets used in this study are sourced from well-established, high-quality open databases, ensuring the reliability of the source data. The closed-source datasets are provided by our partner chemical plants and pharmaceutical companies, consisting of real reaction records, compound manuals, and drug dictionaries that have been carefully curated by domain experts, also ensuring data quality.

For the molecule design, product inference, retrosynthesis prediction, and yield prediction tasks, we use USPTO, CHEBI, CJHIF, and PubChem reaction data for training. We first extract reaction data into reactant SMILES, catalyst SMILES, product SMILES, and yield data. We then apply several cleaning steps to further ensure consistency and usability: (a) We remove invalid or unparsable SMILES strings using RDKit. Reactions missing essential components such as reactants, products, or catalysts are excluded. (b) We discard reactions with structurally abnormal entries, specifically those in which the same compound appears in both the reactants and products. These 2 steps remove 2.1% of invalid entries from the source datasets. For the yield data, the entries are sourced from USPTO and CJHIF. In these datasets, the same chemical reaction may appear multiple times with different yields, corresponding to repeated experiments under identical conditions. Therefore, we average the yield values of identical reactions and use the resulting mean yield for training.

For the molecule description task, we use the Text2Mol and all closed-source datasets for training. We apply the following cleaning procedures: (a) We remove entries with inconsistent mappings between SMILES, IUPAC names, and molecular descriptions. For example, if a SMILES string corresponds to multiple conflicting molecular names or descriptions, the entry is discarded. (b) Identical molecules appearing in multiple sources are deduplicated based on canonical SMILES to prevent training bias toward frequently repeated compounds. (c) All textual descriptions are normalized by removing special characters, correcting typographical errors, and standardizing units and formatting. These steps remove 3.8% of entries from the source datasets.

#### Data augmentation

Then, we jointly conduct 2 data augmentation strategies to obtain 3 samples for the reaction data, for each reaction: (a) permute SMILES for each molecule by RDKit and (b) permute molecule orderings for reactants, catalysts, and products, respectively.

For the molecule description task, we do not apply any additional data augmentation.

#### Prompt construction for instruction tuning

For retro-synthetic prediction, product inference, and yield inference, we organize reactant SMILES, catalyst SMILES, product SMILES, and yield data according to the prompt templates. For molecule design, we use the RDKit tool to randomly select 1 to 20 properties from a candidate pool of 172 properties to fill in the prompt templates. We present prompt templates and examples corresponding to different chemical tasks in Sections S5 and S6.

#### Duplicate removal

We remove exact duplicate reactions based on identical reaction SMILES. However, we do not remove near-duplicate reactions from the training dataset. To assess the potential impact of near-duplicates, we apply Locality-Sensitive Hashing (LSH) with the MinHash algorithm, a widely used method for detecting near-duplicates in large-scale datasets. Specifically, we first encode each reaction into a 1,024-dimensional DRFP [31] vector. Then, we compute a 256-permutation MinHash signature for each reaction and index these signatures using the LSH banding technique to efficiently identify candidate pairs with high similarity. We define near-duplicates as reaction pairs with a Tanimoto similarity greater than 0.95. From a random sample of 100,000 reactions before data augmentation, we identify approximately 1,500 near-duplicate pairs, corresponding to around 1,600 unique reactions. This yields a near-duplicate rate of less than 2%, indicating that the presence of near-duplicates in the training set is minimal and does not significantly affect the final results.

#### Data filtering for safety

To ensure that BatGPT-Chem does not learn or reproduce molecules with potentially harmful properties, we implement a rigorous filtering pipeline aimed at eliminating toxic, addictive, or explosive compounds from the training corpus to the greatest extent possible.

To ensure safety, we conduct an in-depth keyword-based screening of the raw training data. We collect all available hazardous molecules from publicly accessible MSDS (https://www.chemsrc.com/MSDSIndex/) and controlled substances listed in the official drug catalog of the People’s Republic of China (https://www.chemradar.com/tools/cis/inv/648972edbc1d7cdaf02f0acc). These molecules are known to possess toxic, harmful, addictive, or explosive properties, and we explicitly search for their molecular formulas within the training data. In parallel, we perform targeted keyword filtering in molecular annotations and textual descriptions, focusing on terms related to toxicity, harmfulness, addiction, or explosive potential. As a result, only 0.0002% of the entries are identified as containing such terms, and these entries are carefully removed from the dataset before model training.

After constructing the instruction tuning dataset, we apply secondary filtering to ensure that no generated prompts or molecular properties introduce unintended hazardous content. Specifically, we verify that no SMILES sequences are associated with warning keywords or semantic labels indicating risk-related attributes.

Finally, we evaluate the trained model’s behavior by prompting it with queries related to dangerous compound generation. BatGPT-Chem fails to generate or predict molecules with harmful characteristics, confirming that such content is absent from its training distribution. For example, given the prompt: “For a molecule description: ‘It has a role as a toxic and addictive drug.’, the molecule’s potential SMILES code is:”. Since no negative responses are provided during training, the model does not say it cannot generate such a molecule. Instead, it outputs a molecule C(C(CO)O)O, which is a common compound known as glycerine. It is harmless and is frequently used as a moisturizing agent. We conduct 500 such queries and examine the molecules generated by the model. None of the outputs are identified as known toxic, harmful, addictive, or explosive compounds.

We also plan to add a detection mechanism before model input on the trial platform to restrict prompts that may lead the model to generate harmful compounds, in order to maximize safety.

#### Training data details

Table 3 lists the training data scale used for each task. We have over 100 million data entries in total, with an average length exceeding 150 tokens. The total number of tokens trained exceeds 15 billion.

**Table 3.** Training data details

Task	Amount
Retrosynthesis prediction	30,114,006
Product inference	30,114,006
Molecule design	40,695,857
Molecule description	210,469
Yield prediction	10,775,991
Total	111,910,329

### Training details

#### Vocabulary expansion

Since the BatGPT-15B model is originally designed for natural language, particularly Chinese and English, it lacks comprehensive coverage of specialized terms in chemistry or SMILES. Consequently, expanding its vocabulary becomes necessary. We employ the Byte Pair Encoding (BPE) [63] algorithm to train a vocabulary using diverse training data, encompassing various forms of molecular SMILES, chemical equation SMILES expressions, molecular names, and more. We also include all chemical element symbols in the augmented vocabulary to empower the model with the potential to handle all chemical elements. Subsequently, we merge this augmented vocabulary with that of BatGPT-15B, ultimately yielding a final vocabulary size of 151,851.

#### Training settings

We train our model using the deepspeed zero2 strategy on an Nvidia A800 GPU cluster. We set the maximum length to 2,048, the batch size per GPU to 8, utilize the AdamW optimizer with a learning rate of 2 × 10^−4^, and employ the cosine learning rate schedule strategy. We enable gradient checkpointing and set the maximum gradient normalization to 1.0 and the weight decay to 0.1.

#### Training process and convergence

To train BatGPT-Chem, we use substantial computational resources consisting of approximately 16 A800 GPUs running continuously for more than 5 months. Throughout the training, we monitor the loss trajectory and observe stable convergence, with the final training loss reaching approximately 0.2.

## Data Availability

We will release the relevant training code and the curated open-source datasets at https://github.com/yangyifei729/BatGPT-Chem. The released resources will be made available under the Apache 2.0 license, allowing broad use and modification with appropriate attribution. Due to intellectual property and licensing constraints, proprietary training data used during model pretraining cannot be made publicly available.
