# Supplementary material for: BatGPT-Chem: A Foundation Large Model for Chemical Engineering
Source: Research (Wash D C). 2025 Sep 10;8:0827. doi: 10.34133/research.0827 (PMC12421729; doi:10.34133/research.0827)
Supplement: Supplementary 1 — Supplementary Text Figs. S1 to S7 Tables S1 to S3 [file research.0827.f1.pdf]

Supplementary Materials for BatGPT-Chem: A Foundation Large Model For Chemical Engineering

Yifei Yang,[†,‡,¶,#] Runhan Shi,[†,‡,#] Zuchao Li,[§] Shu Jiang,[‖] Bao-Liang Lu,[†,‡] Qibin Zhao,[⊥] Yang Yang,[*,†,‡] and Hai Zhao[*,†,‡,¶]

†*Department of Computer Science and Engineering, Shanghai Jiao Tong University, Shanghai 200240, China*

‡*Key Laboratory of Shanghai Education Commission for Intelligent Interaction and Cognitive Engineering, Shanghai Jiao Tong University, Shanghai 200240, China*

¶*Shanghai Key Laboratory of Trusted Data Circulation and Governance in Web3*

§*National Engineering Research Center for Multimedia Software, School of Computer Science, Wuhan University, Wuhan 430070, China*

‖*School of Artificial Intelligence and Computer Science, Nantong University, Nantong 226019, China*

⊥*RIKEN Center for Advanced Intelligence Project, Tokyo 103-0027, Japan*

#*These authors contributed equally to this work.*

E-mail: yangyang@cs.sjtu.edu.cn; zhaohai@cs.sjtu.edu.cn

Contents

1 Reputable plagiarism check 2

2 Related works 2

3 Additional results **4**

3.1 Ablation study 4

3.2 Additional retrosynthesis prediction comparison 4

3.3 Additional yield prediction comparison 6

4 Retrosynthesis prediction examples **7**

5 Prompt templates **12**

6 Prompt examples **20**

References **23**

1 Reputable plagiarism check

To ensure the originality of our manuscript, we perform a thorough plagiarism check using Turnitin [1]. After excluding our preprint versions previously uploaded to ChemRxiv, the overall similarity with other published papers is 7%. The overlapping content is primarily concentrated in standard descriptions of evaluation datasets, general model introductions, and widely used metric definitions such as MaxFrag and Validity.

We carefully review these sections and address all instances by adding appropriate citations or rephrasing the relevant parts to eliminate any risk of plagiarism.

2 Related works

The advancement of chemistry in recent decades has been closely intertwined with the support of computer and AI technologies. The development of AI applications in the field of chemistry can be broadly categorized into rule-based chemistry AI systems, neural networks and small language model-based chemistry AI systems, and LLM-based chemistry AI

[1] https://www.turnitin.com/

systems.

Rule-based chemistry AI systems. Over the past few decades, there have been numerous rule-based and template-based chemistry AI systems.[1–3] During development, these systems entail significant manual design of reaction templates, involving expert computational chemists, resulting in a challenging design process. They also require users to input compound details and reaction conditions manually, which incurs a high learning cost. Additionally, numerous chemical databases have also been proposed, such as Reaxys, CDS,[4] LIGAND,[5] SciFinder, ChemSpider,[6] and SPRESI which can assist in retrieving various chemical reaction equations, but also require a rather cumbersome usage process and a high learning cost.

Neural network and small language model-based chemistry AI systems. In recent times, there has been a surge in the development of neural network-driven algorithms for chemical prediction,[7–10] marking significant progress in the integration of AI technologies into chemistry. However, these methods are limited in their ability to address a broad spectrum of chemical tasks, focusing instead on specific categories or a narrow range of challenges within the field. Subsequently, there have been some efforts to apply small language models in the field of chemistry, such as PolyBERT[11] and MOLBERT.[12] Similarly, however, these methods are also only capable of addressing a subset of chemical tasks.

LLM-based chemistry AI systems. With LLMs showing immense potential in AI4Science, many studies have also begun to apply LLMs to the field of chemistry. Chemcrow[13] utilizes an LLM-based agent to autonomously plan and execute the syntheses of an insect repellent and three organocatalysts, guiding the discovery of a novel chromophore. A GPT-3-based predictive chemistry approach has been developed,[14] and the broader applications of large language models in chemistry, such as molecular and material property prediction and the design of novel tool interfaces, have also been explored.[15] However, these efforts primarily rely on prompt engineering with existing LLMs, rather than training models tailored for diverse chemical tasks. While a few works have developed chemistry-specific

LLMs, such as ChemDFM[16] and ChemLLM,[17] they face clear limitations that contribute to their suboptimal performance: (1) The scale of their training data is relatively small. Both ChemLLM and ChemDFM are trained on fewer than 2 million samples, whereas BatGPT-Chem is trained on a significantly larger dataset consisting of over 10 million samples, even before data augmentation. This gives BatGPT-Chem a distinct advantage in terms of data richness and diversity, leading to stronger downstream performance. (2) These models rely on general-purpose open-source backbones such as LLaMA[18] and InternLM.[19] In contrast, BatGPT-Chem is built upon our proprietary BatGPT-15B,[20] a foundation model specifically optimized for STEM (Science, Technology, Engineering, Mathematics) domains. This provides better alignment with the knowledge and reasoning demands of chemistry. Additionally, in terms of model size, BatGPT-Chem ranks among the largest chemical foundation models currently available. With 15 billion parameters, it exceeds ChemDFM's 13B model and is second only to the 20B version of ChemLLM. Therefore, BatGPT-Chem is currently among the largest and most powerful bilingual chemical foundation models.

3 Additional results

3.1 Ablation study

To evaluate the impact of decoding strategies and domain-specific training, we conduct an ablation study, as shown in Table S1. Beam search consistently achieves the best performance across all metrics, closely followed by nucleus sampling at different temperatures and Chinese prompts. Notably, BatGPT-15B shows a dramatic performance drop when trained without chemistry-related data, highlighting the critical importance of domain-specific pretraining.

3.2 Additional retrosynthesis prediction comparison

To further benchmark our model, we include comparisons with both a traditional model (Chemformer) and a commercial LLM (GPT-4) in Table S2. The results show that Chem-

Table S1: Top-10 results of BatGPT-Chem with different settings on the USPTO-100 dataset in English, where w/o chemistry denotes the BatGPT-15B model without training on chemistry-related datasets, t is the temperature for nucleus sampling, and l=Chinese is the beam search in Chinese.

Setting	MaxFrag (%)	Coverage (%)	Validity (%)
w/o chemistry	10.0	70.0	87.9
$t = 0.3$	64.0	59.0	**100.0**
$t = 0.5$	**74.0**	68.0	**100.0**
$t = 0.9$	**74.0**	68.0	**100.0**
l =Chinese	70.0	67.0	**100.0**
beam search	**74.0**	**68.4**	**100.0**

former, a task-specific trained model, achieves the highest accuracy and perfect validity. In contrast, LLMs such as GPT-4 and ChemDFM-13B, evaluated in a few-shot or zero-shot setting, perform notably worse in terms of accuracy. Our model achieves higher zero-shot accuracy than other LLMs and maintains 100% validity.

While traditional models remain state-of-the-art in supervised settings, they require task-specific training on downstream data. In contrast, LLMs like BatGPT-Chem provide the benefit of zero-shot prediction, offering greater flexibility and generalization without additional fine-tuning.

Table S2: Top-1 results for retrosynthesis prediction on USPTO-100 dataset. †: results from ChemDFM-13B.[16] *: we omit the catalysts output of BatGPT-Chem since this dataset does not contain.

Model	Accuracy (%)	Validity (%)
Chemformer[21]†	**53.6**	**100.0**
GPT-4 (5-shot)†	11.4	89.0
ChemDFM-13B (0-shot)†	12.0	91.0
BatGPT-Chem (0-shot)*	14.0	**100.0**

3.3 Additional yield prediction comparison

To further assess model performance, we include comparisons with both a traditional model (UAGNN) and a commercial LLM (GPT-4) in Table S3. The results show that UAGNN, a supervised graph-based model, achieves state-of-the-art accuracy across both datasets (SM and HTE BH), outperforming all LLM-based approaches. Other LLMs perform competitively, offering greater flexibility and generalization without additional fine-tuning. Note that large datasets like USPTO and CJHIF contain noisy or inconsistent yield annotations, while high-quality datasets like BH and SM are too small (only thousands of reactions) to effectively train LLMs, potentially limiting their performance.

Table S3: Results (%) for yield prediction on the Suzuki-Miyaura (SM) and the high-throughput experiments Buchwald-Hartwig (HTE BH) datasets. †: results from ChemDFM-13B.

Model	SM	HTE BH
UAGNN[22†]	**95.7**	**96.5**
GPT-4†	76.4	80.0
ChemDFM-13B†	79.3	82.7
BatGPT-Chem	78.9	79.1

4 Retrosynthesis prediction examples

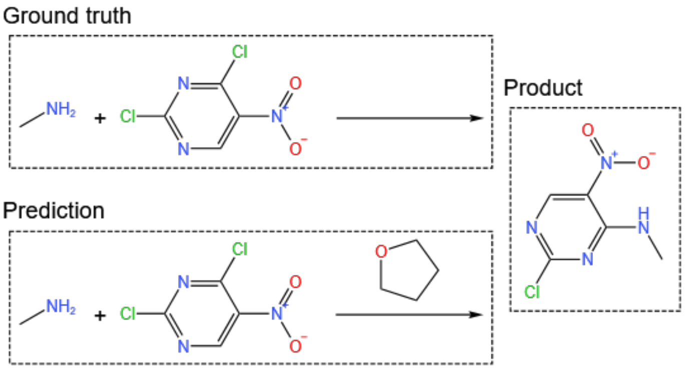

Figure S1: Case 1 from USPTO-50k. Give the product **CNc1nc(Cl)ncc1[N+](=O)[O-]**, the model successfully predicts the correct reactant **CN.O=[N+]([O-])c1cnc(Cl)nc1Cl**. It also simultaneously provides a potential catalyst **C1COCC1**, which is a commonly used catalyst.

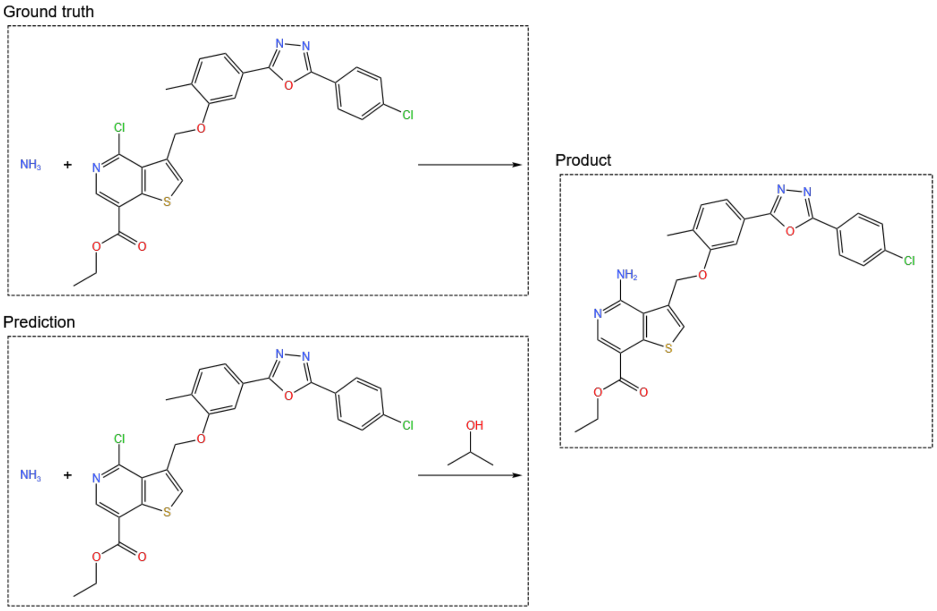

Figure S2: Case 2 from USPTO-50k. Give the product **CCOC(=O)c1cnc(N)c2c(COc3cc(-c4nnc(-c5ccc(Cl)cc5)o4)ccc3C)csc12**, the model successfully predicts the correct reactant **N.Clc1c2c(scc2COc2c(C)ccc(-c3nnc(-c4ccc(Cl)cc4)o3)c2)c(C(OCC)=O)cn1**. The model also predicts a catalyst **C(C)(O)C**, which could act as a solvent.

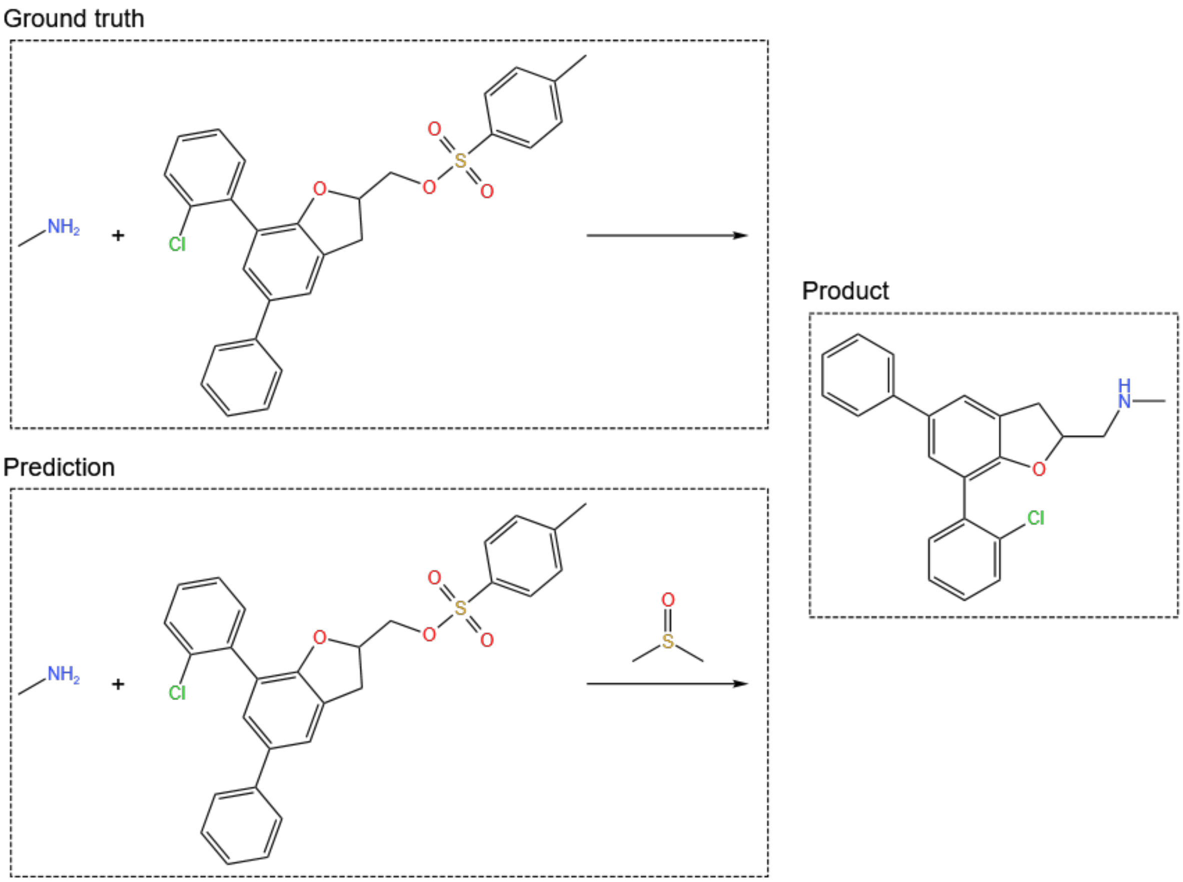

Figure S3: Case 3 from BioChem. Give the product **CNCC1Cc2cc(-c3ccccc3)cc(-c3ccccc3Cl)c2O1**, the model predicts the correct reactant **CN.Cc1ccc(S(=O)(=O)OCC2Cc3cc(-c4ccccc4)cc(-c4ccccc4Cl)c3O2)cc1**. A catalyst **S(C)(=O)C**, which bears a resemblance to the reactant structure, is predicted, possibly serving as a “reaction fragment” or an “intermediate product”.

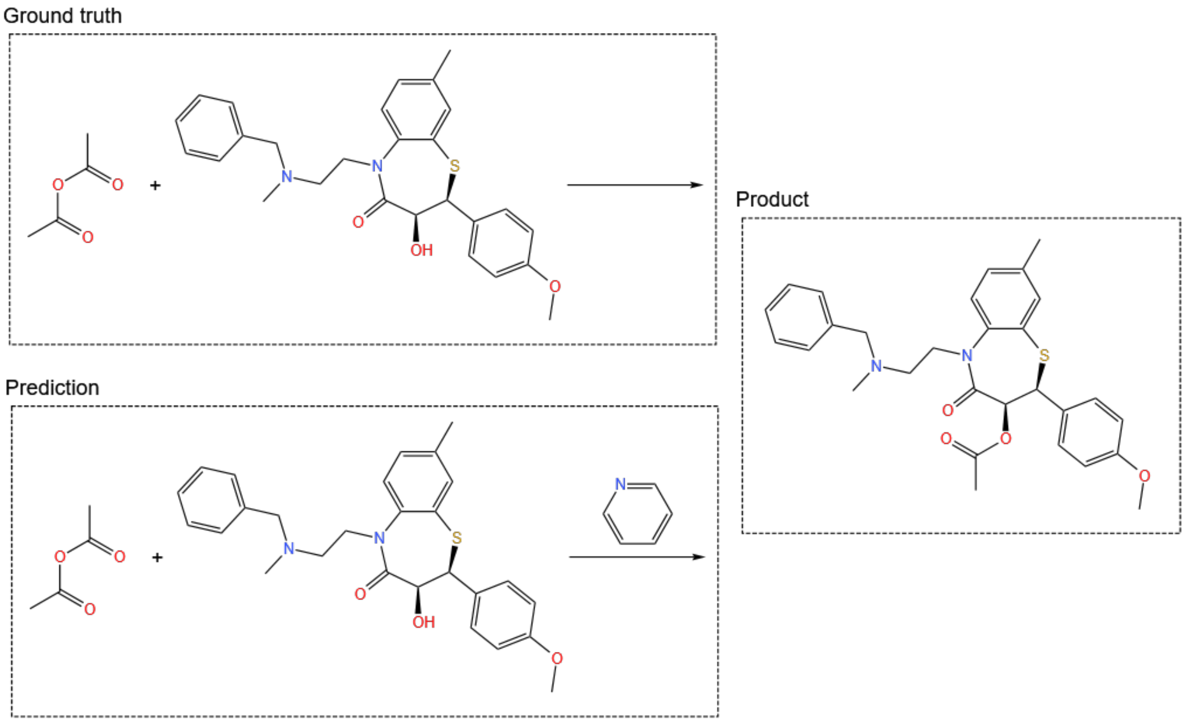

Figure S4: Case 4 from BioChem. For the product **COc1ccc([C@@H]2Sc3cc(C)ccc3N(CCN(C)Cc3ccccc3)C(=O)[C@@H]2OC(C)=O)cc1**, the model successfully predicts the reactant **CC(=O)OC(C)=O.c12ccc(C)cc1S[C@@H](c1ccc(OC)cc1)[C@@H](O)C(=O)N2CCN(C)Cc1ccccc1** and provides a catalyst **c1cccnc1**, which could be a solvent.

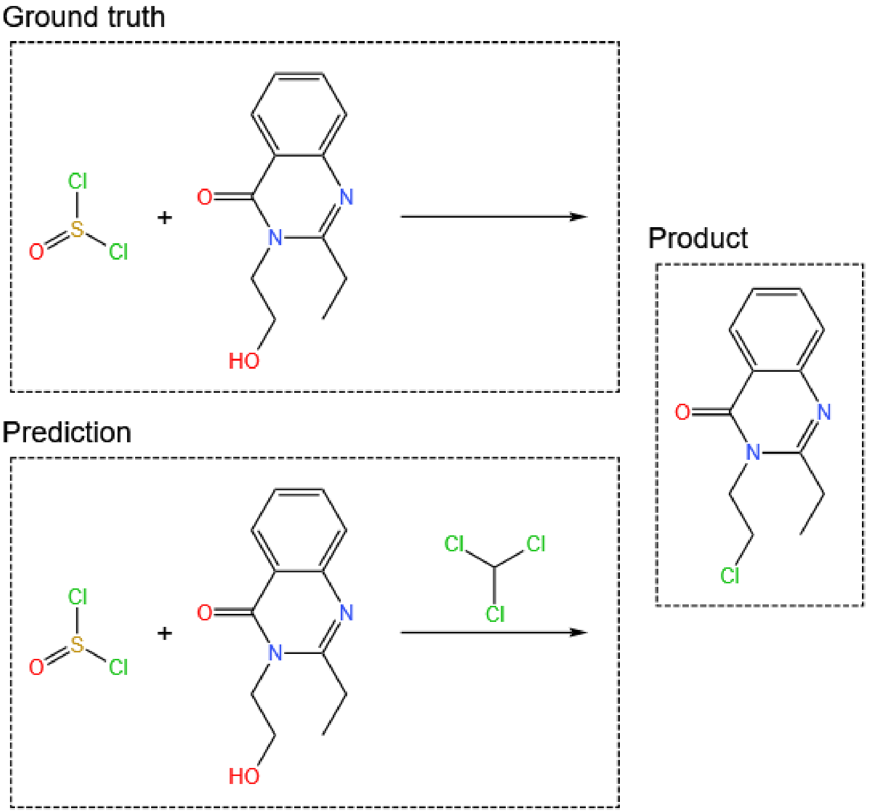

Figure S5: Case 5 from BioChem. For the product **CCc1nc2ccccc2c(=O)n1CCCl**, the model predicts the correct reactants **O=S(Cl)Cl.c12ccccc1nc(CC)n(CCO)c2=O**, and provides a catalyst **ClC(Cl)Cl**, which could be a solvent.

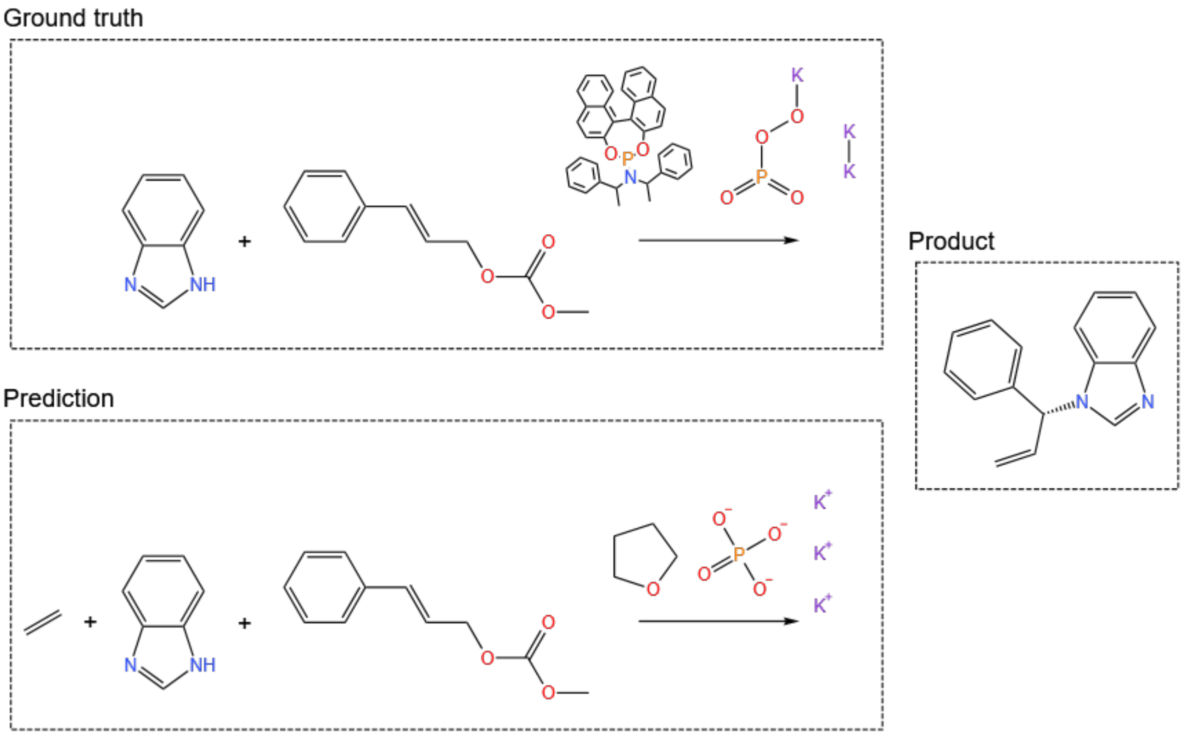

Figure S6: Case 6 from our private benchmark AAAA. For the product **C=C[C@H](c1ccccc1)n1cnc2ccccc21**, the model predicts a reactant **C=C.c12ccccc1[nH]cn2.O=C(OC/C=C/c1ccccc1)OC** with an additional small molecule ethylene, and successfully predicts furan as the solvent. The solvent information appears only in the original paper of this reaction, demonstrating that the model successfully transfers knowledge from the literature to retrosynthesis prediction tasks after being trained on a large dataset.

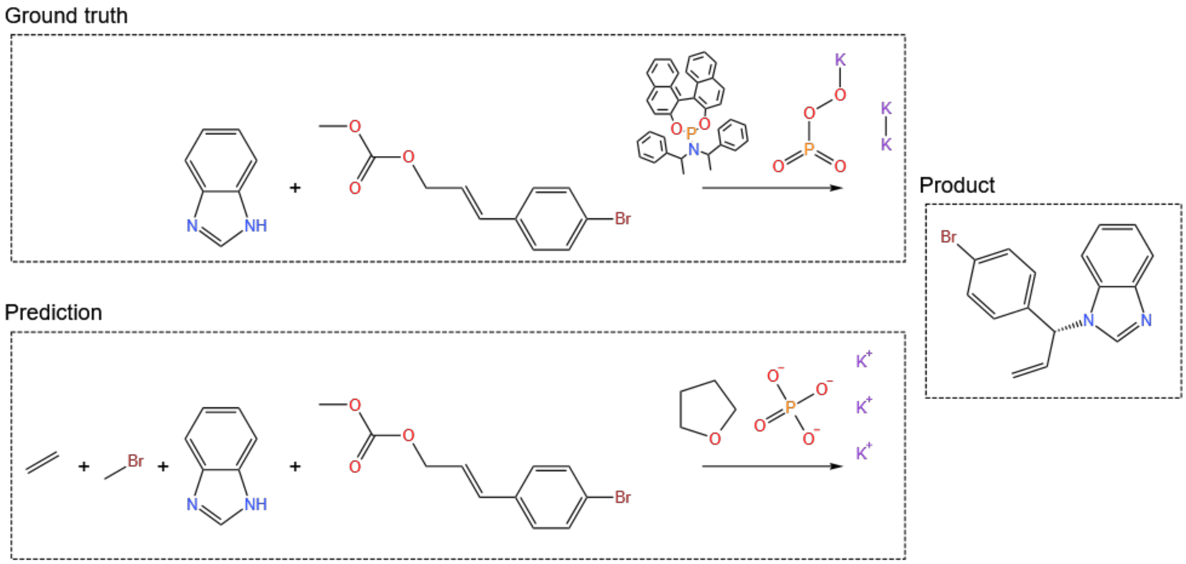

Figure S7: Case 7 from our private benchmark AAAA. For the reactant **C=C[C@H](c1ccc(Br)cc1)n1cnc2ccccc21**, the model correctly predicts the reactant **c1nc2ccccc2[nH]1.CBr.C=C.COC(=O)OC\\C=C\\c1ccc(Br)cc1** but includes an additional ethylene and its corresponding hydrogen halide molecule. The main reason might be the presence of a halogen substituent on the reactant's ring, which is relatively reasonable. The ligand is not predicted, but furan is successfully predicted as the solvent.

5 Prompt templates

Chemical Task	Subtask	Chinese Prompt Template	English Prompt Template
Retrosynthesis Prediction	Reactant and Catalyst Prediction	1. 给定产物的SMILES码为{}，对应的反应物和催化剂可能为：<br><br>2. 已知产物的SMILES码为{}，相应的反应物和催化剂可能为：<br><br>3. 给定产物的SMILES码为{}，可以推断出潜在的反应物和催化剂为：<br><br>4. 对于SMILES码为{}的产物，相应的反应物和催化剂可能为：<br><br>5. 现有产物，其SMILES码为{}，则用于合成它们的反应物和催化剂可能为：	1. Given the SMILES codes of the products , the corresponding reactants and the catalysts can be:<br>2. Knowing the SMILES codes of the products, the corresponding reactants and catalysts can be:<br>3. Given the SMILES codes {} of the products, potential reactants and catalysts can be deduced as:<br>4. With the SMILES codes of the products provided, potential reactants and catalysts can be deduced as follows:<br>5. Given the SMILES codes {} of the products, potential reactants and catalysts can be inferred as:
	Reactant Prediction	1. 给定产物的SMILES码{}和催化剂的SMILES码{}，可能的反应物包含：<br>2.给定产物的SMILES码{}和催化剂的SMILES码{}，潜在的反应物可能包括：<br><br>3.当一个化学反应的产物的SMILES码为{}和催化剂的SMILES码为{}时，可能的反应物有：<br><br>4.当反应得到的产物的SMILES码为{}，催化剂为{}，可能的反应物包含：<br><br>5.对于SMILES码为{}的产物和SMILES码为{}的催化剂，这个反应可能的反应物是：	1. Given the SMILES codes of the products {} and the catalysts {}, the possible reactants can be:<br>2. Provided with the SMILES codes of the products {} and the catalysts {}, potential reactants may include:<br>3. When given the SMILES codes of the products {} and the catalysts {}, the potential reactants could be:<br>4. When provided with the SMILES codes of the products {} and the catalysts {}, potential reactants to consider are:<br>5. With the SMILES codes of the products {} and the catalysts {} provided, potential reactants may encompass:

Chemical Task	Subtask	Chinese Prompt Template	English Prompt Template
Product Inference	Product and Catalyst Prediction	1. 给定反应物的SMILES码为{}，可以与之搭配的催化剂以及反应得到的产物为：<br><br>2. 现有反应物的SMILES码包括{}。可以与它们搭配的催化剂以及得到的产物为：<br><br>3. 对于SMILES码为{}的反应物，可以在反应时加入的催化剂以及得到的产物为：<br><br>4. 使用的反应物的SMILES码为{}，可以加入的催化剂和得到的产物可能为：<br><br>5. 对于反应物{}，潜在的可以加入的催化剂以及对应得到的产物可能为：	1. The SMILES codes of the reactants are {}. The corresponding catalysts it can pair with and the resulting products are:<br>2. The existing reactants include {}. The catalysts that can be matched with them and the resulting products are as follows:<br>3. The given reactants are {}. The catalysts that can be combined with them, along with the resulting products, are as follows:<br>4. With the SMILES codes of the products {} provided, potential reactants and catalysts can be deduced as follows:<br>5. With the specified reactants as {}, the associated catalysts and the resulting products can be:
	Product Prediction	1. 给定反应物的SMILES码为{}，催化剂的SMILES码为{}，产物为：<br>2. 反应物的SMILES码为{}，使用的催化剂的SMILES码为{}，则反应产生的产物为：<br>3. 当给定的反应物的SMILES码为{}，催化剂的SMILES编码为{} 时，产物为：<br>4. 当反应物{} 加入催化剂{} 进行反应的时候，得到的产物为：<br>5.{}表示反应物的SMILES编码，{}表示催化剂，反应得到的产物为:	1. Given the SMILES codes of the reactants {}, the catalysts {}, the products are:<br>2. With the SMILES codes of the reactants {} and the catalysts {}, the resulting products are:<br>3. When the SMILES codes of the reactants {} and the catalysts {} are given, the products are:<br>4. The reactants {} and the catalysts {} will determine the resulting products:<br>5. The SMILES codes of the reactants are {}, the catalysts are {}, and the products are:

Chemical Task	Subtask	Chinese Prompt Template	English Prompt Template
	Specify Catalyst Molecular Properties	1. 要求用反应物{}制备产物{}，要求催化剂满足条件：{}，催化剂可以是：<br><br>2. 实现由反应物{}合成产物{}的过程需要考虑满足条件：{}的催化剂，催化剂可以是：<br><br>3. 由反应物{}合成产物{}的过程，选用的催化剂需要满足条件：{}，催化剂可以被选择为：<br><br>4. 为了使反应物{}合成产物{}，可选择一种满足条件：{}的催化剂，催化剂可以是：<br><br>5. 用反应物{}制备产物{}，可以添加催化剂，要求其符合条件：{}，催化剂可为：	1. Given reactants {} and products {}, the catalysts are required that meets the conditions: {}, so the catalysts can be:<br>2. Provided with reactants {} and products {}, the catalysts needed must satisfy the conditions: {}. Possible catalysts include:<br>3. Given reactants {} and products {}, the catalysts required should meet the specified conditions: {}. Potential catalysts may be:<br>4. With specified reactants {} and products {}, the catalysts needed can meet the conditions: {}. Potential catalysts can be:<br>5. Given the specified reactants {} and products {}, the catalysts required have the capability to satisfy the conditions: {}. Possible catalysts include:
Molecular Design	Specify Reactant and Catalyst Molecular Properties	1. 为了制备产物{}，要求反应物满足条件：{}，催化剂满足条件：{}，反应物和催化剂分别可以是：<br><br>2. 要合成产物{}，反应物要满足：{}的条件，而催化剂也需要满足：{}的要求，反应物和催化剂分别可以是：<br><br>3. 实现产物{}的合成过程需要考虑满足条件：{}的反应物，以及满足条件：{}的催化剂，则反应物和催化剂可以是：<br><br>4. 为了合成产物{}，要求反应物满足:{}的条件，催化剂满足：{}的条件，则可以选择的反应物和催化剂分别是：<br><br>5.要合成产物{}，选用的反应物需要满足条件：{}，催化剂需要满足条件：{}，则可以选择：	1. To synthesize the products {}, it is required that the reactants meet the conditions: {}, and the catalysts satisfy the conditions: {}. The reactants and the catalyst can be:<br>2. To synthesize the products {}, the reactants need to meet the conditions: {}, and the catalysts also need to satisfy the requirements: {}. The reactants and the catalysts can be:<br>3. The synthesis process for the products {} involves considering reactants that meet the conditions: {}, as well as the catalysts that satisfy the requirements: {}. The reactants and the catalysts can be::<br>4. To synthesize the products {}, it is required that the reactants meet the conditions: {}, and the catalysts satisfy the conditions: {}. The possible choices for reactants and catalysts are:<br>5.To synthesize the products {}, the chosen reactants need to meet the conditions: {}, and the catalysts should satisfy the conditions: {}. The selection can include:

Chemical Task	Subtask	Chinese Prompt Template	English Prompt Template
Molecular Design	Specify Reactant, Catalyst and Product Molecular Properties	1. 对于一个可以进行的化学反应，要求反应物满足条件：{}，催化剂满足条件：{}，产物满足条件：{}，则这个反应的反应物、催化剂和产物分别可以是：<br>2. 对于一个可发生的化学反应，要求反应物符合条件：{}，同时催化剂要满足条件：{}，产物也需要符合条件：{}。这个反应的具体反应物、催化剂和产物分别可以是：<br>3. 进行一种可行的化学反应时，要求反应物满足条件：{}，催化剂符合条件：{}，产物符合条件：{}。这个反应所涉及的反应物、催化剂和产物分别可以是：<br>4. 在进行某一可实施的化学反应时，反应物的选择需要满足条件：{}，催化剂也需要符合条件：{}，得到的产物满足条件：{}。这个反应中的具体反应物、催化剂和产物分别可以是：<br>5. 对于一种可进行的化学反应，反应物满足条件：{}，催化剂满足条件：{}，产物满足条件：{}。则所选的反应物、催化剂和对应产物可以是：	1.For a feasible chemical reaction, the reactants meet the conditions: {}, the catalysts satisfy the conditions: {}, and the products fulfill the conditions: {}. The specific reactants, catalysts, and products for this reaction can be:<br>2. For a possible chemical reaction, it is required that the reactants meet the conditions: {}, and the catalysts satisfy the conditions: {}, while the products also fulfill the conditions: {}. The specific reactants, catalysts, and products for this reaction can be:<br>3. When conducting a feasible chemical reaction, it is required that the reactants meet the conditions: {}, the catalysts should satisfy the conditions: {}, and the products comply with the conditions: {}. The specific reactants, catalysts, and products involved in this reaction can be:<br>4. When conducting a feasible chemical reaction, the choice of reactants needs to meet the conditions: {}, the catalysts also satisfy the conditions: {}, and the resulting products fulfill the conditions: {}. The specific reactants, catalyst, and product involved in this reaction can be:<br>5. For a possible chemical reaction, the reactants meet the conditions: {}, the catalysts meet the conditions: {}, and the products comply with the conditions: {}. The chosen reactants, catalysts, and the corresponding products can be:

Chemical Task	Subtask	Chinese Prompt Template	English Prompt Template
Molecular Description	Given the Chinese name of the drug, generate an English name and description	1. 给定一个药物的中文名称{}，它的对应英文名称和一些相关描述是：<br>2. {}这种药物对应的英文名和一些相关描述如下所示：<br>3. 对于{}这一药物，其英文名和一些药物描述如下：<br>4. 以下是关于{}药物对应的英文名称和一些药物描述：<br>5.一个药物的中文名称是{}，它的英文名和药物描述是：	
	Given the English name of the drug, generate an Chinese name and description	1. 英文名为{}的药物的中文名和药物描述如下：<br>2. 英文名为{}的药物对应的中文名和一些相关描述为：<br>3. 给出{}这种药物的中文名和一系列相关的药物描述：<br>4. 以下是关于{}药物对应的中文名称和一些药物描述：<br>5.一个药物的英文名称是{}，它的中文名和药物描述是：	
	Given the drug description, generate Chinese and English names	1. 药物描述为{}对应的药物中文名和英文名分别是：<br>2. 一个药物的描述为{}，那么它可能的中文名称和英文名称分别是：<br>3. 如果药物的描述为{}，那么可能的中文名称和英文名称是：<br>4.{}这个描述对应的药物的中文名和英文名称可能是：<br>5. 如果有一个药物被描述为{}，那么它的可能中文名称和英文分别是：	
	Given the molecular name, generate the molecular formula	1. 给定一个分子名{}，它对应的分子式为：<br>2. 一个分子的名字是{}，其相应的分子式是：<br>3. 以{}为名字的分子，其分子式是：<br>4. 对于一个以{}为名字的分子，其分子式是：<br>5. {} 所代表的分子的分子式是：	
	Given the molecular formula, generate the molecular name	1. 一个分子的分子式是 {}，它的名字是：<br>2. 分子式为 {}的分子对应的名字是：<br>3. {}分子式对应的分子名是：<br>4. 对于分子式{}，它的名字为：<br>5. {} 分子式的名称是：	

Chemical Task	Subtask	Chinese Prompt Template	English Prompt Template
	Given the molecular description, generate molecular IUPAC name and SMILES code		1. Given a description of a molecule: {}, the possible IUPAC name and corresponding SMILES code for this molecule are:<br>2. When provided with a molecule description: {}, the potential IUPAC name and corresponding SMILES code for the molecule can be determined as:<br>3. In the context of a molecule description: {}, the molecule's potential IUPAC name and its corresponding SMILES code are:<br>4. In the case of a molecule described as: {}, the molecule's possible IUPAC name and the corresponding SMILES code can be:<br>5. A molecule described as: {} may have a potential IUPAC name and corresponding SMILES code:
Molecular Description	Given the molecular SMILES code, generate molecular IUPAC name and description		1. Given a SMILES code of a molecule: {}, the possible IUPAC (International Union of Pure and Applied Chemistry chemical nomenclature) name and corresponding description for this molecule are:<br>2. If given a SMILES code representing a molecule as {}, the potential IUPAC (International Union of Pure and Applied Chemistry chemical nomenclature) name and related description can be:<br>3. In the case of a molecule with the SMILES code {}, the potential IUPAC name and description for the molecular are:<br>4. When provided with a SMILES code {} for a molecule, the IUPAC (International Union of Pure and Applied Chemistry chemical nomenclature) name and associated description for the compound can be:<br>5. Given a SMILES code {} for a molecule, the potential IUPAC name and its related description can be identified as:

Chemical Task	Subtask	Chinese Prompt Template	English Prompt Template
Molecular Description	Given the molecular IUPAC name, generate molecular description and SMILES code		1. Given a IUPAC (International Union of Pure and Applied Chemistry chemical nomenclature) name of a molecule: {}, the possible SMILES code and corresponding description for this molecule are:<br>2. The SMILES code and description for the molecule with the IUPAC (International Union of Pure and Applied Chemistry chemical nomenclature) name {} are:<br>3. Providing the IUPAC (International Union of Pure and Applied Chemistry chemical nomenclature) name {} for a molecule, the possible SMILES code and accompanying description are:<br>. Given the IUPAC (International Union of Pure and Applied Chemistry chemical nomenclature) name {} for a molecule, the potential SMILES code and its corresponding description are:<br>5. Providing the IUPAC name {} for a molecule, the potential SMILES code and a corresponding description can be:
Yield Prediction		1. 对于一个化学反应，其反应物的SMILES码 {}，催化剂 {}，产物 {}，期望的产率值是：<br>2. 对于一个反应物的SMILES码{}，催化剂{}，产物{}的化学反应，期望的产率值为：<br>3. 一个化学反应的反应物、催化剂和产物的SMILES码分别为{}、{}、{}，其期望产率是<br>4. 为了使反应物{}合成产物{}，可选择一种满足条件：{}的催化剂，催化剂可以是：<br>5. 对于一个反应物的SMILES码为 {}、催化剂的SMILES码为 {} 、产物的SMILES码为 {} 的化学反应，其期望产率是：	1. Given the SMILES codes of the reactants {}, the catalysts {}, the products {}, the expected yield value of this chemical reaction is:<br>2. By examining the SMILES codes of the reactants {}, the catalysts {}, and the products {}, the expected yield value of this chemical reaction can be estimated as:<br>3. Through an examination of the SMILES codes of the reactants {}, the catalysts {}, and the products {}, the expected yield value of this chemical reaction is:<br>4. The reactants, catalysts, and products of a chemical reaction are {}, {} and {}, and its expected yield is:<br>5. The reactants {}, catalysts {}, and products {} define a chemical reaction, and its expected yield is:

6 Prompt examples

Chemical Task	Subtask	Chinese Prompt	English Prompt
	Reactant and Catalyst Prediction	给定产物的SMILES码为COc1ccccc1OCCN1CC(COc2ccc3c(c2[nH]c2ccccc32)OCC1=O，可以推断出潜在的反应物和催化剂为：	Given the product SMILES codes COc1ccccc1OCCN1CC(COc2ccc3c(c2)[nH]c2ccccc32)OCC1=O, the corresponding reactants and catalysts can be:
Retrosynthesis Prediction	Reactant Prediction	1. 给定产物的SMILES码CC(=C)c1cccc(C)c1Br和催化剂的SMILES码[K+]， CC(C)(C)[O-]和C1CCOC1，可能的反应物包含：<br>2.（不指定催化剂）给定产物的SMILES码CC(=C)c1cccc(C)c1Br，潜在的反应物可能包括：	1. Given the SMILES codes of the products CC(=C)c1cccc(C) c1Br and the catalysts [K+], CC(C) (C)[O-] and C1CCOC1, the possible reactants can be:<br>2. (No catalyst specified) Provided with the SMILES codes of the products CC(=C)c1cccc(C)c1Br, potential reactants may include:
	Product and Catalyst Prediction	给定反应物的SMILES码为C(c1ccccc1)Br, c1(C)ccccc1, C(C)(=O)C 和 C=C，可以与之搭配的催化剂以及反应得到的产物为：	The SMILES codes of the reactants are C(c1ccccc1)Br, c1(C)ccccc1, C(C)(=O)C and C=C. The corresponding catalysts it can pair with and the resulting products are:
Product Inference	Product Prediction	1. 给定反应物的SMILES码为O=CN,O(C(=O)C(OC(C)=O)N1C(CC#C[Si](C)(C)C)CC1=O)C(C)C, C=C和COC(C)=O，催化剂的SMILES码为C(Cl)Cl和Cl[Sn](Cl)(Cl)Cl，产物为:<br>2. （不指定催化剂）反应物的SMILES码为c1c(F)cc(F)c(N)c1和c1(CCCCn2nncc2)ccc(cc1)OCc1occ(C(O)=O)n1，则反应产生的产物为：	1. Given the reactant SMILES codes O=CN, O(C(=O)C(OC(C)=O)N1C(CCC#C[Si](C)(C)C)CC1=O)C(C)C, C=C and COC(C)=O, the catalysts C(Cl)Cl and Cl[Sn](Cl)(Cl)Cl, the products are:<br>2. (No catalyst specified) With the reactant SMILES codes Cl, C1OCCOC1 and n1c2c([nH]c1-c1c(I)ccnc1OC)cc(C#N)cc2C, the resulting products are:
Molecular Design	Specify Catalyst Molecular Properties	用反应物 [Na+], c1(C(c2ccc(O)cc2)=O)ccccc1, [H-], C(Br)C(CO)(CO)CBr 和 C(Br)C1(CO)COC1 制备产物 O=C(C1=[CH][CH]=[CH][CH]=[CH]1)C1=[CH][CH]=C(O[CH2]C2([CH2][OH])[CH2]O[CH2]2)[CH]=[CH]1，可以添加催化剂，要求其符合条件：杂原子的数量≥ 0.0 并且< 2.4, 酰胺的数量≥ 0.0 并且<0.7, 氢键受体的数量≥ 0.0 并且 < 1.4，催化剂可为：	Given reactants CO, COC, NC=O and COc1ccccc1OCCN(CC(O)COc1ccc2c(c1)[nH]c1ccccc21)C(=O)CCl and products COc1ccccc1OCCN1CC(COc2ccc3c(c2)[nH]c2ccccc32)OCC1=O, the catalysts required should meet the specified conditions: the number of Heteroatoms ≥ 0.0 and < 2.4, the number of Hydrogen Bond Donors ≥ 0.0 and < 1.9, the total number of NHs or OHs ≥ 0.0 and < 2.5, the number of Hydrogen Bond Acceptors ≥ 0.0 and < 1.4, Wildman-Crippen LogP value ≥ -4.1 and < -0.1, the exact molecular weight of the molecule ≥ 0.0 and < 204.6, Wildman-Crippen MR value ≥ 0.0 and < 52.1. Potential catalysts may be:

Chemical Task	Subtask	Chinese Prompt	English Prompt
	Specify Reactant and Catalyst Molecular Properties	要合成产物[CH3]C([CH3])([CH3])OC(=O)N1[CH2][CH]=C(N([CH2]C2=[CH][CH]=[CH][CH]=[CH]2)C(=O)C2=C(I)[CH]=[CH][CH]=[CH]2)[CH2][CH2]1，反应物要满足：分子的价电子数≥179.0 并且<286.8, 分子的NH和OH总数量≥2.5 并且<5.5, 卤素原子的数量≥1.0 并且<3.3, 旋转键的数量≥0.0 并且 <6.0, 羰基氧原子的数量≥ 1.6 并且 <3.6, Wildman-Crippen MR值≥129.0 并且 <205.9 的条件，而催化剂也需要满足：分子的准确分子量≥0.0 并且 <204.6, 卤素原子的数量≥0.0 并且 <1.0, 氢键供体的数量≥0.0 并且 <1.9, 分子的氮氧原子总数量≥0.0 并且<1.3 的要求，反应物和催化剂分别可以是：	To synthesize the products [CH3]C([CH3])([CH3])OC(=O)N1[CH2][CH]=C(N([CH2]C2=[CH][CH]=[CH][CH]=[CH]2)C(=O)C2=C(I)[CH]=[CH][CH]=[CH]2)[CH2][CH2]1, the reactants need meet the conditions: the number of amides ≥0.7 and <2.1, the number of ether oxygens (including phenoxy) ≥0.0 and <1.5, the number of Hydrogen Bond Acceptors ≥ 1.4 and <6.1, the total number of NHs or OHs ≥2.5 and <5.5, the number of Hydrogen Bond Donors ≥0.0 and <1.9, Balaban's J value ≥-0.4 and <0.8, the number of heavy atoms ≥32.7 and <52.5, and the catalysts also need to satisfy the requirements: Balaban's J value ≥1.9 and <3.1, the number of Hydrogen Bond Acceptors ≥0.0 and <1.4, the number of benzene rings ≥0.0 and <1.6. The reactants and the catalysts can be:
Molecular Design	Specify Reactant, Catalyst and Product Molecular Properties	进行一种可行的化学反应时，要求反应物满足条件：Wildman-Crippen MR值≥ 129.0 并且 < 205.9, 分子的价电子数≥ 179.0 并且 < 286.8, 醚氧原子的数量（包括苯氧基）≥ 0.0 并且 < 1.5, BertzCT值≥ 146.5 并且 < 925.1, 氢键供体的数量≥ 0.0 并且 < 1.9, Balaban's J值≥ -0.4 并且 < 0.8, 分子的准确分子量≥ 493.6 并且 < 782.5, 酰胺的数量≥ 0.7 并且 < 2.1, 分子的NH和OH总数量≥ 2.5 并且 < 5.5, 氢键受体的数量≥ 1.4 并且 < 6.1，催化剂符合条件：Wildman-Crippen LogP值≥ -0.1 并且 << 3.8, Balaban's J值≥ 1.9 并且 < 3.1, Wildman-Crippen MR值≥ 0.0 并且 < 52.1, 分子的重原子数≥ 0.0 并且 < 13.0, 酰胺的数量≥ 0.0 并且 < 0.7, 分子的准确分子量≥ 0.0 并且 < 204.6, 氢键受体的数量≥ 0.0 并且 <1.4, 氢键供体的数量≥ 0.0 并且 < 1.9, 分子的氮氧原子总数量≥ 0.0 并且 < 1.3, 醚氧原子的数量（包括苯氧基）≥ 0.0 并且 < 1.5，产物符合条件：氢键受体的数量≥ 1.4 并且 < 6.1, 酰胺的数量≥ 0.7 并且 < 2.1, 苯环的数量≥ 1.6 并且 < 3.3, 分子的准确分子量≥ 493.6 并且 < 782.5, Wildman-Crippen MR值≥ 52.1 并且 < 129.0。这个反应所涉及的反应物、催化剂和产物分别可以是：	When conducting a feasible chemical reaction, it is required that the reactants meet the conditions: the number of ether oxygens (including phenoxy) ≥0.0 and <1.5, the number of heavy atoms ≥32.7 and <52.5, the total number of Nitrogens and Oxygens ≥1.3 and <7.1, the number of valence electrons the molecule ≥179.0 and <286.8, the total number of NHs or OHs ≥2.5 and <5.5, the number of Hydrogen Bond Acceptors ≥1.4 and <6.1, the catalysts should satisfy the conditions: the number of Hydrogen Bond Acceptors ≥0.0 and <1.4, Balaban's J value ≥1.9 and <3.1, Wildman-Crippen MR value ≥0.0 and <52.1, the number of carbonyl O ≥0.0 and <1.6, the number of valence electrons the molecule ≥ 0.0 and <71.3, the total number of Nitrogens and Oxygens ≥0.0 and <1.3, the number of amides ≥0.0 and <0.7, Wildman-Crippen LogP value ≥-0.1 and <3.8, the number of halogens atoms ≥0.0 and <1.0, the number of Heteroatoms ≥0.0 and <2.4, and the products comply with the conditions: the number of amides ≥0.7 and <2.1, the total number of NHs or OHs ≥0.0 and <2.5, the exact molecular weight of the molecule ≥493.6 and <782.5. The specific reactants, catalysts, and products involved in this reaction can be:

Chemical Task	Subtask	Chinese Prompt	English Prompt
	Given the drug Chinese name, generate English name and description	给定一个药物的中文名：先锋哌唑酮，那么它的对应英文名称和一些相关描述是	
	Given the drug English name, generate Chinese name and description	一个药物的英文名称是 Ped-el，它的中文名和药物描述是：	
	Given the drug description, generate Chinese and English names	一个药物的描述为 [类别]镇静催眠抗惊厥药,[适应症] 用于治疗神经衰弱、忆病、神经性失眠、精神兴奋状态。 [用量用法] 口服:每次10ml,1日3次。 [注意事项] 1.不宜用于浮肿和少尿及癫痫病人。 2.其他参见溴化钾及溴化铵。 [规格] 溶液:含溴化钾3%、溴化钠3%、溴化铵3%。 那么它可能的中文名称和英文名称分别是：	
Molecular Description	Given the molecular name, generate its molecular formula	给定一个分子名 trideuteriomethyl 2,2,2-tribromoacetate，它对应的分子式为：	
	Given the molecular formula, generate its molecular name	分子式为 C21H14K4N2O9的分子对应的名字是：	
	Given the molecular description, generate molecular IUPAC name and SMILES code		In the context of a molecule description: 'It has a role as an antimanic drug. It is an inorganic chloride and a lithium salt.', the molecule's potential IUPAC (International Union of Pure and Applied Chemistry chemical nomenclature) name and its corresponding SMILES code are:
	Given the molecular SMILES code, generate molecular IUPAC name and description		Given a SMILES code '[Cl-].[K+]' for a molecule, then the potential IUPAC name and its related description can be identified as:
	Given the molecular IUPAC name, generate molecular description and SMILES code		Given a IUPAC (International Union of Pure and Applied Chemistry chemical nomenclature) name of a molecule: 'magnesium;dichloride;hydrate', the possible SMILES code and corresponding description for this molecule are:
Yield Prediction		对于一个化学反应，其反应物的SMILES码 CCl.CBr.Brc1ccc(cc1)C(=O)CN(=O)=O.ON=C(Cl)c1cccc(Cl)c1，催化剂 CCN(CC)CC.CO，产物 Clc1cccc(c1)c1onc(c1N(=O)=O)-c1ccc(Br)cc1，期望的产率值是：	Given the molecular formulas of the reactants CCl.CBr.Brc1ccc(cc1)C(=O)CN(=O)=O.ON=C(Cl)c1cccc(Cl)c1, the catalysts CCN(CC)CC.CO, the products Clc1cccc(c1)-c1onc(c1N(=O)=O)-c1ccc(Br)cc1, the expected yield value is:

References

(1) Corey, E. J.; Wipke, W. T. Computer-Assisted Design of Complex Organic Syntheses. *Science* **1969**, *166*, 178–192.

(2) Smith, S. G.; Sherwood, B. A. Educational Uses of the PLATO Computer System: The PLATO system is used for instruction, scientific research, and communications. *Science* **1976**, *192*, 344–352.

(3) Lu, G.; Ai, S.; Li, J. Layer-by-layer assembly of human serum albumin and phospholipid nanotubes based on a template. *Langmuir* **2005**, *21*, 1679–1682.

(4) Fletcher, D. A.; McMeeking, R. F.; Parkin, D. The United Kingdom chemical database service. *Journal of chemical information and computer sciences* **1996**, *36*, 746–749.

(5) Goto, S.; Nishioka, T.; Kanehisa, M. LIGAND: chemical database for enzyme reactions. *Bioinformatics (Oxford, England)* **1998**, *14*, 591–599.

(6) Ayers, M. ChemSpider: the free chemical database. *Reference reviews* **2012**, *26*, 45–46.

(7) Fooshee, D.; Mood, A.; Gutman, E.; Tavakoli, M.; Urban, G.; Liu, F.; Huynh, N.; Van Vranken, D.; Baldi, P. Deep learning for chemical reaction prediction. *Molecular Systems Design & Engineering* **2018**, *3*, 442–452.

(8) Xie, X.; Clark Spotte-Smith, E. W.; Wen, M.; Patel, H. D.; Blau, S. M.; Persson, K. A. Data-driven prediction of formation mechanisms of lithium ethylene monocarbonate with an automated reaction network. *Journal of the American Chemical Society* **2021**, *143*, 13245–13258.

(9) Schwaller, P.; Vaucher, A. C.; Laino, T.; Reymond, J.-L. Prediction of chemical reaction yields using deep learning. *Machine learning: science and technology* **2021**, *2*, 015016.

(10) Meuwly, M. Machine learning for chemical reactions. *Chemical Reviews* **2021**, *121*, 10218–10239.

(11) Kuenneth, C.; Ramprasad, R. polyBERT: a chemical language model to enable fully machine-driven ultrafast polymer informatics. *Nature Communications* **2023**, *14*, 4099.

(12) Fabian, B.; Edlich, T.; Gaspar, H.; Segler, M.; Meyers, J.; Fiscato, M.; Ahmed, M. Molecular representation learning with language models and domain-relevant auxiliary tasks. 2020.

(13) Bran, A. M.; Cox, S.; White, A. D.; Schwaller, P. Chemcrow: Augmenting large-language models with chemistry tools. *arXiv preprint arXiv:2304.05376* **2023**,

(14) Jablonka, K. M.; Schwaller, P.; Ortega-Guerrero, A.; Smit, B. Leveraging large language models for predictive chemistry. *Nature Machine Intelligence* **2024**, 1–9.

(15) Jablonka, K. M.; Ai, Q.; Al-Feghali, A.; Badhwar, S.; Bocarsly, J. D.; Bran, A. M.; Bringuier, S.; Brinson, L. C.; Choudhary, K.; Circi, D.; others 14 examples of how LLMs can transform materials science and chemistry: a reflection on a large language model hackathon. *Digital Discovery* **2023**, *2*, 1233–1250.

(16) Zhao, Z.; Ma, D.; Chen, L.; Sun, L.; Li, Z.; Xu, H.; Zhu, Z.; Zhu, S.; Fan, S.; Shen, G.; others ChemDFM: Dialogue Foundation Model for Chemistry. *arXiv preprint arXiv:2401.14818* **2024**,

(17) Zhang, D.; Liu, W.; Tan, Q.; Chen, J.; Yan, H.; Yan, Y.; Li, J.; Huang, W.; Yue, X.; Zhou, D.; others ChemLLM: A Chemical Large Language Model. *arXiv preprint arXiv:2402.06852* **2024**,

(18) Touvron, H.; Lavril, T.; Izacard, G.; Martinet, X.; Lachaux, M.-A.; Lacroix, T.; Rozière, B.; Goyal, N.; Hambro, E.; Azhar, F.; others Llama: Open and efficient foundation language models. *arXiv preprint arXiv:2302.13971* **2023**,

(19) Cai, Z. et al. InternLM2 Technical Report. *CoRR* **2024**, *abs/2403.17297*.

(20) Li, Z.; Zhang, S.; Zhao, H.; Yang, Y.; Yang, D. Batgpt: A bidirectional autoregessive talker from generative pre-trained transformer. *arXiv preprint arXiv:2307.00360* **2023**,

(21) Irwin, R.; Dimitriadis, S.; He, J.; Bjerrum, E. J. Chemformer: a pre-trained transformer for computational chemistry. *Machine Learning: Science and Technology* **2022**, *3*, 015022.

(22) Kwon, Y.; Lee, D.; Choi, Y.-S.; Kang, S. Uncertainty-aware prediction of chemical reaction yields with graph neural networks. *Journal of Cheminformatics* **2022**, *14*, 1–10.
